# Label-Free Degradation Diagnosis for Classifier Selection in Hyperspectral Scenes

**DOI:** 10.3390/s26185837

**Published:** 2026-09-15

**Authors:** Cagri Kaymak, Cem Atilgan

**Affiliations:** 1 Department of Mechatronics Engineering, Faculty of Engineering, Firat University, 23119 Elazig, Türkiye; 2 Department of Mechatronics Engineering, Faculty of Technology, Kirklareli University, 39020 Kirklareli, Türkiye; cematilgan@klu.edu.tr

**Keywords:** hyperspectral image classification, label-free degradation diagnosis, classifier robustness, degradation structure, spatial data leakage, intrinsic dimensionality

## Abstract

**Highlights:**

**What are the main findings?**

- A two-layer, label-free diagnosis performed before classification reports the magnitude of degradation and separates four degradation structures, correctly classifying all fifty-six structure-labelled operating points across seven evaluation scenes.
- At a matched severity, degradation within the existing bands is more harmful than adding the same variance as separate bands, and classifier fragility follows two opposing regimes.

**What are the implications of the main findings?**

- Classifier selection should be guided by the diagnosed noise regime, since no classifier family remains robust across all degradation structures and regime-based selection produced the better map in every case examined.
- Robustness claims should be interpreted together with the evaluation protocol, as random pixel splitting can inflate accuracy by up to 0.249 and change the best classifier in six of the ten scenes.

**Abstract:**

In hyperspectral image classification, the most suitable classifier depends on the magnitude and the type of degradation present in the scene; this information, however, cannot be obtained without labels at the stage where the choice has to be made. A two-layer diagnosis computed before classification and without labels is proposed in this study: a severity index reports the magnitude of the degradation, while three scene-derived indicators separate independent, band-dependent, spectrally correlated and striped structures from one another, while classification itself remains supervised. The approach is evaluated on ten benchmark scenes under a leakage-aware spatial protocol. Classifier fragility is found to follow two opposing regimes: tree ensembles are robust to spatially local degradation but fragile to degradation spread across the spectrum, whereas the opposite is observed for the convolutional network. Regime-based classifier selection produces the better classification map in every combination examined. Replacing the spatial protocol with a random pixel split is further shown to inflate accuracy by between 0.162 and 0.249 and to change the best classifier in six of the ten scenes.

## 1. Introduction

Hyperspectral images record hundreds of contiguous, narrow spectral bands for every pixel. This property enables materials to be discriminated in detail in applications ranging from precision agriculture and mineralogy to environmental monitoring and urban mapping [1,2]. This spectral richness also brings a very high nominal feature dimensionality, which is generally regarded as a disadvantage for supervised classification. The classical justification is the curse of dimensionality: as the number of features grows relative to the number of labelled training samples, the classifier accuracy first rises and then falls. This peaking behavior was formalized by Hughes [3] and has been observed repeatedly in remote sensing studies.

Two geometric mechanisms are usually held responsible: the concentration of pairwise distances, which erodes the contrast between the nearest and the farthest neighbor of a query point [4,5], and hubness, whereby a few points dominate the neighbor lists of the rest [6]. Both degrade the distance-based reasoning and, together, encourage the almost universal use of dimensionality reduction in hyperspectral workflows, from principal component analysis and band selection [7] to manifold learning [8,9,10] and deep feature learning [11,12].

In parallel with this widespread use of dimensionality reduction, deep-learning methods for hyperspectral classification have also advanced rapidly in recent years. The trend began with convolutional neural networks [13,14], was extended to capsule architectures designed for limited training samples [15], and continued with transformer-based architectures [16,17,18], attention-based recurrent networks [19], graph convolutional networks [20], graph-structure-aware transformers [21], morphological attention transformers [22] and, most recently, state-space Mamba models [23]. A comprehensive review of the transformer-based branch of this literature is also available [24]. Although these methods achieve a high accuracy, most of them treat high nominal dimensionality as the problem and do not separate the effect of dimensionality from the effect of noise in a controlled manner.

The failure to separate dimensionality from noise raises the question of how the degradation itself is addressed. Robustness has recently begun to be examined explicitly in the hyperspectral literature, but from a different direction. Chen et al. [25] evaluated seven convolutional classifiers under simulated degradations within a mutation analysis framework and ranked them by the robustness they retained. Chandrappa, Paheding, and Reyes-Angulo [26] showed that the adversarial robustness of vision transformers depends strongly on the spatial patch size, with smaller patches remaining more stable. Similarly, Nalepa et al. [27] simulated on-board acquisition conditions and reported how deep segmentation models respond to them. The reference-free assessment is not new in hyperspectral imaging either: Yang et al. [28] estimate the image quality without a clean reference by learning quality-sensitive statistics. The aim of the approach proposed here is different; the objective is not the perceptual quality of the image but the type of degradation present and which classifier family that degradation endangers. The studies in question measure robustness afterwards, that is, by corrupting data whose degradation is known at the time of construction and observing how a particular model responds. The complementary question addressed here is whether the degradation in a scene can be characterized before classification and without labels, so that the classifier selection can be guided by this information rather than audited afterwards. The same distinction also applies to the rapid architectural development in the field, where transformers and state-space models have come to the fore [29,30,31,32,33]: a diagnosis computed from the data alone is independent of the architecture that is eventually selected.

It is useful to state what existing methods can and cannot provide at the point where the classifier has to be chosen. The reference-free image quality assessment estimates the perceptual quality without a clean reference [25], and noise estimation methods report a noise level per band or per scene; both therefore address the magnitude, and neither identifies the structure of the degradation. Restoration and denoising methods characterize the structure implicitly, since a striping-removal method presupposes striping, but they do so in order to remove it rather than to report it, and the choice of method already encodes the answer. Robustness benchmarks report which classifier degrades least under a given corruption [22,24], which is precisely the information needed, but they obtain it by training and evaluating classifiers on labelled data after the corruption is known. What is missing is a measurement that reports both the magnitude and structure from the scene alone, before any classifier is trained, and that maps onto a classifier decision. This is the gap the present study addresses.

Whether the degradation needs to be characterized before classification depends on what actually causes the loss of accuracy. At this point, there is a critical assumption that is rarely tested in practice. Because neighboring wavelengths measure overlapping physical phenomena, hyperspectral bands are strongly correlated; the intrinsic dimensionality of a scene, in other words, the number of degrees of freedom the spectra actually occupy, is therefore usually far smaller than the nominal number of bands. A similar low-dimensional structure has also been documented for natural images [34]. If this is the case, the true cause of the accuracy loss may not be nominal dimensionality; what is measured as a dimensionality effect when components are added and removed may, in fact, reflect the amount of uninformative, noise-like spectral variance that those components carry. Sweeps that heuristically vary the number of components cannot separate these two explanations, because they change the dimensionality and informativeness at the same time; a controlled test of which of the two drives the degradation is missing from the literature. The joint effect of dimensionality, noise, and limited training sets was also noticed in early kernel-based hyperspectral studies, in which the classifier accuracy was assessed jointly under noisy conditions, high input dimensionality, and small training sets [35]. That study, however, varied these factors simultaneously and compared classifiers under the resulting conditions; the question of which factor is responsible for the loss, therefore, remained open. The present study departs precisely at this point: dimensionality and noise are varied independently of each other at an equal number of bands, so that their contributions are determined separately rather than jointly. In addition, whether the degradation is added as separate bands or mixed into the existing bands is treated as an independent variable that previous studies did not isolate.

This uncertainty has a practical consequence. Which method should be preferred in hyperspectral classification depends on how much and what type of degradation the scene contains; this information, however, is not available at the moment the choice has to be made. The effect of the degradation normally becomes visible only after the classifiers have been trained and accuracy has been measured on labelled test pixels, that is, long after the decision stage. Since labelled data are the scarcest resource in hyperspectral applications, an indicator able to recognize degradation before classification and without labels could turn method selection from trial and error into measurement.

In response to these two gaps, a two-layer diagnosis computed before classification and without labels is proposed. The severity index computed from distance concentration reports the magnitude of the degradation, while three scene-derived indicators report its type. The distinction on which the diagnosis rests was established through a controlled experiment: ambient dimensionality was separated from noise at an equal number of bands, and adding the same variance as separate bands was then compared, at a matched severity, with mixing it into the existing bands. This second comparison is necessary because real sensor degradation forms on top of the existing bands rather than adding new ones. The finding that the second case is more harmful shows that the effective quantity is not only the amount of uninformative variance the scene carries but where that variance is placed. The diagnosis was therefore designed to report not only the magnitude of the degradation but also its structure. The decision value of the diagnosis was established through the behavior under noise of six classifiers from different families. Because the spatial dependence between neighboring and nearly identical pixels inflates the accuracy in random pixel splits, leakage-aware spatial splits were used in all comparisons [36,37]. That classifier robustness under noise is method-dependent and that random pixel splits inflate reported accuracy are already recognized in the hyperspectral literature [25,27]; what this study adds is their magnitude under a controlled design, together with the two findings on the degradation structure that follow from it. The term label-free refers throughout to the diagnosis and to the model selection step, not to the classification itself: the six classifiers compared here are supervised methods trained on labelled pixels. What the diagnosis avoids is the use of labels to decide which classifier to apply, a decision that would otherwise require training and evaluating several classifiers on labelled test pixels before any of them could be chosen.

The contributions of this study are as follows:

(1) Ambient dimensionality and uninformative variance are separated at an equal number of bands, and in-band degradation is shown to be more harmful than added bands at matched severity.
(2) Classifier fragility is shown to follow two opposing regimes: tree ensembles are robust to spatially local degradation, the convolutional network to degradation spread across the spectrum.
(3) A two-layer diagnosis computed before classification and without labels is proposed, reporting both the magnitude and the structure of the degradation, and is used to select a classifier family.
(4) The evaluation protocol is treated as an experimental variable and is shown to determine the ranking of the classifiers, not only the reported accuracy level.

The remainder of the paper is organized as follows. Section 2 describes the severity and structure indicators, the two-layer diagnostic rule built from them, the datasets, the classifiers, and the leakage-aware evaluation protocol. Section 3 presents, first, the precondition of the diagnosis, and then the classifier fragility, the predictive power of the indicators, the underlying mechanism, and the decision value of the diagnosis. Section 4 discusses the findings and the limitations of the study, and Section 5 summarizes the main conclusions.

## 2. Materials and Methods

### 2.1. Severity Index

The severity index is based on distance concentration and is computed in several steps. In the first step, all pairwise Euclidean distances are computed with Equation (1) for n pixels drawn at random from the standardized feature space. Here $x_{i}$ and $x_{j}$ denote the spectral vectors of pixels i and j, D the number of bands, and $d_{ij}$ the distance between the two pixels. A total of n(n−1)/2 distances is obtained:

(1) $$d_{ij}={\left\|x_{i}-x_{j}\right\|}_{2}=\sqrt{\sum_{b=1}^{D}{\left(x_{i}^{\left(b\right)}-x_{j}^{\left(b\right)}\right)}^{2}}$$

In the second step, the mean of these distances is obtained with Equation (2) and their standard deviation with Equation (3):

(2) $$\mu_{d}=\frac{1}{M}\sum_{i<j}d_{ij}$$

(3) $$\sigma_{d}=\sqrt{\frac{1}{M}\sum_{i<j}{\left(d_{ij}-\mu_{d}\right)}^{2}}$$

In the third step, the relative spread of the distance distribution is measured by the ratio of the standard deviation to the mean. As dimensionality or noise increases, the distances concentrate and this ratio decreases. The severity index is defined by subtracting this ratio from one, as in Equation (4), so that higher values correspond to stronger concentration. Because the index is a transformation of the coefficient of variation of the distances, it can, in principle, take negative values:

(4) $$L_{conc}=1-\frac{\sigma_{d}}{\mu_{d}}$$

Subtracting the ratio of intrinsic dimensionality to the nominal number of bands from one defines the redundancy measure in Equation (5). Here, $d_{int}$ denotes the intrinsic dimensionality:

(5) $$L_{red}=1-\frac{d_{int}}{D}$$

All of these quantities are computed before classification, without labels, on a random subsample of pixels. Relative contrast and a hubness measure based on the skewness of the k-occurrence distribution are also reported as complementary diagnostic variables [6]. This index is an adaptation of established concentration diagnostics based on the coefficient of variation of distances to hyperspectral scenes, as a pre-classification and label-free severity measure [4,5]. Although fractional norms are known to concentrate less severely [5], the Euclidean norm was used deliberately. This is because the purpose of the index is not to identify the best-behaved metric but to measure the degradation of the metric that the affected classifiers actually use: both the k-nearest neighbor method and the RBF kernel operate on Euclidean distances.

### 2.2. Structure Indicators

The severity index measures the magnitude of the degradation but does not separate its type; classifier selection, however, depends on the structure of the noise. Three complementary indicators that can be computed before classification and without labels are therefore proposed: adjacent-band correlation targets correlated noise, band-variance spread targets band-dependent signal-to-noise ratio, and the striping score targets column-wise striping. All three indicators are evaluated on a random subsample of at most twenty thousand pixels, and a constant $\varepsilon =10^{-12}$ is added to every denominator so that the quantities remain finite. The order of operations is, moreover, decisive for the interpretation of the indicators. The bands of the undisturbed scene are, first, centred and scaled to unit variance, the degradation is added to this standardized cube, and the indicators are computed on the resulting cube without rescaling. In this arrangement, the band-variance spread measures the extent to which variances that were unit before the degradation have become unequal, and, by design, it remains close to zero in an undisturbed scene. Because the scaling of a new scene cannot be estimated before the degradation, this indicator has to be computed on raw radiance. Adjacent-band correlation and the striping score, in contrast, are directly reference-free and require no undisturbed reference scene.

Expressed in notation, $z_{i}^{\left(b\right)}$ denotes below the z-scored value of pixel i in band b among the n sampled pixels, ${\sigma_{b}}^{2}$ the pixel variance of the same band, and C the matrix of column profiles obtained by centring the cube along the rows, with one row per image column and one column per band. Adjacent-band correlation is defined by Equation (6) as the mean of the absolute empirical correlation between consecutive band pairs over the D−1 pairs:

(6) $$C_{adj}=\frac{1}{D-1}\sum_{b=1}^{D-1}\left|\frac{1}{n}\sum_{i=1}^{n}z_{i}^{\left(b\right)}z_{i}^{\left(b+1\right)}\right|$$

The band-variance spread is the standard deviation across bands of the natural logarithm of the per-band variances and is given by Equation (7). Variance was used rather than standard deviation, and the logarithm was taken to base e:

(7) $$V_{spr}={std}_{b}\left(ln\left(\sigma_{b}^{2}+\varepsilon \right)\right)$$

The striping score is the product of two factors. The first is the column-structure ratio, the mean over bands of the ratio of the column-profile variance of each band to the pixel variance of that band; $c_{b}$ denotes the column of the matrix C corresponding to band b. The second is one minus the shared ratio; the shared ratio $p_{1}$ is the proportion of variance explained by the first principal component after the columns of C have been standardised band-wise. This distinction is necessary: genuine spatial column structure is shared across bands, in other words a bright column is bright in every band and $p_{1}$ approaches one, whereas stripe noise is independent in each band. The product in Equation (8) therefore remains small unless the column structure is both present and unshared.

(8) $$S_{str}=\left(\frac{1}{D}\sum_{b=1}^{D}\frac{var\left(c_{b}\right)}{\sigma_{b}^{2}+\varepsilon }\right)\left(1-p_{1}\right)$$

Beyond the synthetic evaluation of the structure indicators, a complementary reference-free measure was tested on real sensor degradation. The raw versions of the Indian Pines and Salinas scenes contain 220 and 224 bands, respectively. In both cases, twenty bands are conventionally removed because of atmospheric water absorption and low signal-to-noise ratio. These are spectral regions in which imaging spectrometers are known to deliver little usable signal [38] and, therefore, constitute a labelled degradation set based on community consensus. In this test, the spatial residual signal-to-noise ratio, estimated from horizontal neighboring-pixel differences within each band, was used. The radiance recorded by a hyperspectral sensor carries thermal, quantization, and shot noise, in addition to atmospheric effects [39]; local spatial residuals provide a simple estimate of this noise. Because standardizing the bands beforehand would, by definition, remove band-dependent signal-to-noise differences, this indicator has to be computed on raw radiance.

### 2.3. Two-Layer Diagnostic Rule

**Figure 1:**
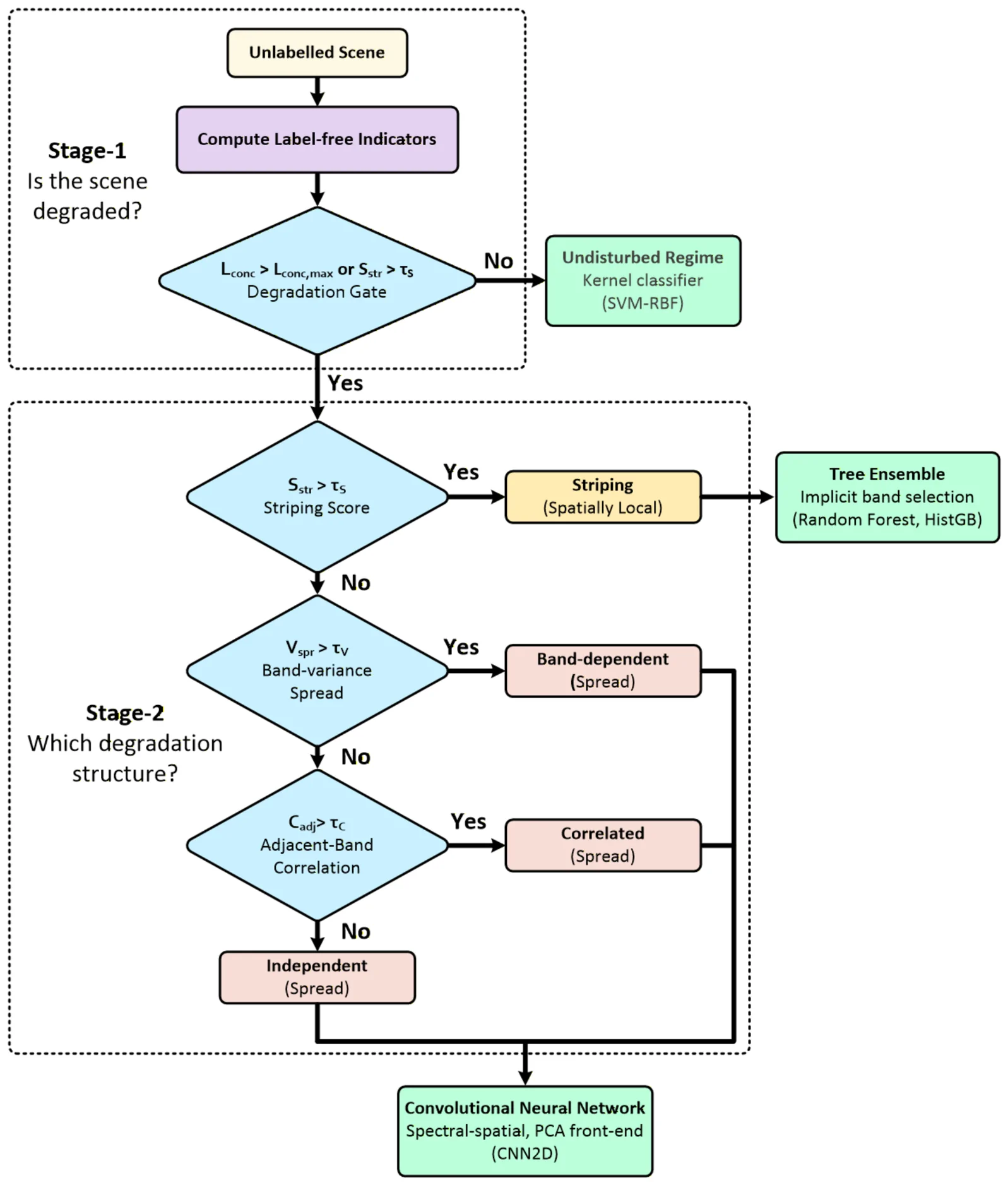
The regime-based selection rule.

The indicators defined in the previous two subsections are converted into a decision rule that guides classifier selection in two stages. The first stage is a binary decision, referred to hereafter as the gate, that determines whether appreciable degradation is present in the scene. The second stage is applied only to scenes that pass the gate and assigns the degradation to one of four structures. This separation is necessary because the structure indicators measure not the presence of degradation but its type, and they also produce a structure label when applied to an undisturbed scene. The complete rule is shown as a flowchart in Figure 1.

The gate is built on two quantities. A scene is regarded as degraded when the severity index exceeds the highest value observed in the undisturbed data of the calibration scenes, or when the striping score exceeds its own threshold. The two-part structure is necessary because the two quantities respond to different physical effects: adding variance raises the severity index whereas removing signal lowers it, and deadlines instead appear as unshared column structure. For the severity threshold the upper bound of the undisturbed data was preferred over the midpoint between the positive and negative class medians; this choice aims to capture milder degradations without sacrificing specificity. The behavior of the gate on the evaluation scenes is reported in Section 3.2.

In scenes that pass the gate, the type of degradation is determined by applying the three structure indicators as a sequential rule: a striping score above its threshold indicates striping; otherwise, a band-variance spread above its threshold indicates band-dependent signal-to-noise ratio; failing that, an adjacent-band correlation above its threshold indicates correlated noise; in all other cases, the degradation is treated as independent noise. The numerical thresholds were not set by hand but derived by taking the midpoint between the positive and negative class medians for each indicator on three calibration scenes, namely, Indian Pines, Pavia University, and Salinas. Confidence intervals were obtained by bootstrap resampling with two thousand repetitions, and the values are reported in Section 3.2. The remaining seven scenes were evaluated with the numerical thresholds never adapted on them.

The output of this procedure, referred to as the regime-based selection rule, is one of two cases. In scenes that do not pass the gate, a clean-regime policy in the form of SVM–RBF, fixed from the three calibration scenes, is applied, since per-scene selection would require labels. In scenes that pass the gate, a classifier is selected from the family corresponding to the assigned structure: a tree ensemble for spatially local degradation and the convolutional network for degradation spread across the spectrum. Band-dependent signal-to-noise ratio is assigned to the spread regime, since it affects every band without producing spatial structure; both families are comparatively robust to this structure, as the in-band degradation results in Section 3.2.2 show, so the assignment follows the definition of the regime rather than a performance difference. What the rule selects is a classifier family together with its preprocessing, not an individual classifier: the two tree ensembles behave almost identically across all six degradation structures, and the front end of the convolutional network is part of its architecture rather than a separate choice. Section 3.3 reports separately how much of the robustness of each family originates from preprocessing.

The rule targets four degradation structures. Two further structures examined in the robustness experiments fall outside its definition but are still handled in practice. Deadlines are detected by the striping component of the gate, since removed columns appear as unshared column structure, and are therefore routed to the tree ensemble, which which the in-band degradation results in Section 3.2.2 show to be the appropriate family for that structure. The mixed structure may fall into any branch depending on the weights of its components; extending the rule to mixtures is left to future work.

The order in which the components of the rule were fixed is stated explicitly here, since it determines whether the procedure is prospective or retrospective. The three calibration scenes, Indian Pines, Pavia University, and Salinas, were designated before any threshold was computed. The numerical thresholds were then derived on those three scenes alone and were never recomputed, adjusted, or verified on the seven evaluation scenes. The mapping from degradation regime to classifier family was likewise fixed from the in-band degradation experiment, in which the family with the lower mean loss under each regime was taken, and was not revised after the evaluation scenes were processed. The evaluation scenes therefore contribute no information to either the thresholds or the mapping; they are used only to measure how a rule that was fixed beforehand performs. The one departure from this order is documented in Section 4.2: the striping indicator was revised after its first version failed on three scenes with strong natural column structure, so the evaluation of that indicator is not as independent as that of the other two.

### 2.4. Datasets and Preprocessing

Ten publicly available hyperspectral scenes were used and are summarized in Table 1: Indian Pines, Pavia University (PaviaU), Pavia Centre (PaviaC), Salinas, Kennedy Space Center (KSC), Botswana, the HyRank Dioni and Loukia scenes, and the Houston 2013 and Houston 2018 scenes. Only labelled pixels were retained and classes with fewer than fifteen labelled pixels were removed. Each band was then scaled to zero mean and unit variance; in the classification experiments, this scaling was learned only on the training pixels of each split and subsequently applied to the test pixels. Transfer of information from the test distribution into training was thereby prevented. The severity index, in contrast, being a label-free and classifier-independent diagnostic measure, was computed on a random subsample of pixels drawn from the entire scene. The intrinsic dimensionality of each scene was further determined with the Levina–Bickel maximum likelihood estimator [40]. The Houston 2013 and Houston 2018 scenes were harmonized for this study as follows: From the original 349 × 1905 pixel, 144-band version of Houston 2013, the 48 bands in the 0.38–1.05 micrometer range corresponding to the 48 bands of Houston 2018 were extracted, and the 209 × 955 pixel area where the two scenes overlap was used. The labels were further reduced to the seven land-cover classes common to both scenes: healthy grass, stressed grass, trees, water, residential buildings, non-residential buildings, and road. This reduction follows from the class definitions of the two scenes overlapping only in these seven classes and is unrelated to the removal of classes with fewer than fifteen labelled pixels. As a result, 2530 labelled pixels were obtained for Houston 2013 and 53,200 for Houston 2018. The ten scenes are divided into two disjoint groups that are used consistently throughout the study. Three scenes, Indian Pines, Pavia University, and Salinas, form the calibration group: the numerical thresholds of the diagnostic rule are derived on these scenes, and the map-level demonstration of Section 2.9 also uses them. The remaining seven scenes, Pavia Centre, Kennedy Space Center, Botswana, Dioni, Loukia, Houston 2013, and Houston 2018, form the evaluation group and are never used to set any threshold or mapping. The fifty-six structure-labelled operating points referred to in the results are the combinations of these seven evaluation scenes, the four degradation structures targeted by the rule, and the two injection levels, that is, 7 × 4 × 2. The corresponding calibration group contains twenty-four points, that is, 3 × 4 × 2.

### 2.5. Classifiers

Six classifiers from different families were evaluated: k-nearest neighbor (KNN); the support vector machine with a radial basis function kernel (SVM–RBF), a strong baseline method in hyperspectral classification [35,41]; a random forest with 200 trees, whose behavior on hyperspectral data with limited training samples has been examined in detail [42]; histogram-based gradient boosting (HistGB), a histogram-binned variant of gradient boosting [43,44]; the multilayer perceptron (MLP) trained by error backpropagation [45]; and a two-dimensional spectral–spatial convolutional neural network (CNN2D) that follows the principal component plus two-dimensional convolution design introduced for hyperspectral data by [13], within the broader CNN-based deep feature extraction paradigm established by Chen et al. [46]. Such spectral–spatial convolutional models remain competitive baselines today [2,47]. The convolutional network used here reduces the spectrum to twenty principal components fitted only on the training pixels, extracts 9 × 9 spatial patches around each pixel, and follows two convolution blocks with global average pooling and a linear classifier. In terms of implementation, the classical models were implemented with scikit-learn 1.8.0 [48] and the convolutional network with PyTorch 2.6.0 [49], both under Python 3.13.9. Such patch-based convolutional designs have also been compared systematically on agricultural scenes [50], and the wider space of spectral–spatial feature extraction strategies has been reviewed recently [51]. The six families were selected to span different decision mechanisms rather than to represent the current state of the art, because the subject under examination is not the accuracy ranking of architectures but how a mechanism responds to uninformative or structured variance. The set accordingly spans a distance-based rule, a kernel margin, two axis-aligned ensembles that perform implicit feature selection, a fully connected network that uses all input dimensions, and patch-based spectral–spatial convolution. Current transformer and state-space architectures are, moreover, further examples of the patch-based spectral–spatial family in this classification, since they take the spatial patch as input and reduce the spectrum through a learned embedding [26,31]. A further point is that each classifier was used with standard parameters and without scene-specific tuning, so that the measured differences reflect the mechanism rather than unequal tuning effort. The comparison was, therefore, made between mechanisms under controlled degradation rather than between methods published on undisturbed benchmarks; the ranking that such benchmarks produce is additionally examined in Section 3.4. The implementation details are as follows: k=5 for KNN; C=100 and scaled gamma for SVM–RBF; 200 trees for the random forest; (128, 64) hidden layers, ReLU activation, the Adam optimiser, and, at most, 300 iterations for the MLP; and library defaults for HistGB. The convolutional network consists of two 3 × 3 convolution blocks (32 and 64 filters, batch normalization and ReLU), global average pooling, and a linear layer, and was trained with the Adam optimizer, a learning rate of $10^{-3}$, 20 epochs, a batch size of 64, and cross-entropy loss. Intrinsic dimensionality was estimated by varying the neighbor count k between 10 and 20, and the severity index was computed on a subsample of 1500 pixels. Finally, in order to determine whether the robustness of the convolutional network arises from its architecture or from the principal component front end, a version of each classical classifier operating on the same twenty-component front end was also evaluated; this front end was likewise fitted only on the training pixels. No hyperparameter was selected using the evaluation protocol. The values listed above are library defaults or values in common use for these classifiers, and they were fixed before any accuracy was measured; no scene-specific tuning, grid search, or validation split was performed, and the test pixels were never consulted in setting them. This choice is deliberate rather than a simplification: tuning each classifier per scene would confound the mechanism under study with unequal tuning effort, and the comparison here is between decision mechanisms under controlled degradation rather than between tuned implementations. The cost of this choice is that the absolute accuracies are lower than those obtainable with per-scene tuning. Its robustness is examined in Section 3.4, where the convolutional network is evaluated over a grid of principal component counts and patch sizes and the variation across that grid is shown to be of the same order as the variation between repeated splits.

### 2.6. Leakage-Aware Evaluation

In hyperspectral benchmark scenes, the labelled pixels are spatially clustered. Random pixel splits, therefore, place nearly identical neighboring pixels into the training and test sets together and inflate the reported accuracy [36,37]. This concern is particularly relevant when few labelled samples are available, a regime that has received dedicated attention [52]. To prevent this effect, a spatial split protocol was used in all evaluations. For each class, the training and test pixels were separated along a spatial axis, and a buffer band as wide as the patch width of the convolutional network was left so that the receptive fields of the training and test samples do not overlap. At most, 150 training samples per class and a fifty per cent test share were further used, each condition was repeated over ten independent splits, and the results are reported as means with standard deviations. In line with the same principle, scaling, the principal component transform, and all other data-dependent operations were fitted only on the training pixels of each split. Overall accuracy (OA), average accuracy, and the Kappa coefficient were recorded as performance measures [53]. In order to determine how strongly the reported accuracy depends on this protocol choice, the entire evaluation on undisturbed data was additionally repeated using a stratified random pixel split instead of the leakage-aware spatial split, with everything else held fixed: the same ten scenes, the same six classifiers, the first five of the ten splits used elsewhere in the study generated with the same seeds, the same upper limit of 150 training pixels per class, and the same upper limit on test set size. The only difference between the two runs is the way labelled pixels are assigned to training and test; every difference in accuracy can therefore be attributed to the protocol alone. The result is reported in Section 3.4.

The split is constructed as follows, so that it can be reproduced exactly: For each class, the labelled pixels of that class are ordered by their column coordinate, and a single cut point is placed so that the pixels on one side form the training pool and those on the other the test pool; the cut is chosen per class rather than per scene, so that every class contributes to both pools and no class is lost. A buffer strip is then removed from both sides of the cut. Its width equals the patch width of the convolutional network, which is nine pixels in all reported experiments, so that no training patch and no test patch can share a pixel; the same buffer is applied to the pixel-based classifiers even though they do not use a spatial neighborhood, so that all six classifiers see identical training and test pools. From the training pool, at most 150 pixels per class are drawn, and the test pool is capped at half of the remaining labelled pixels of that class. The ten repetitions differ only in the random draw within the pools and in the position of the cut point, both controlled by a seed; the seeds are fixed across scenes, classifiers, and degradation conditions, so that any two conditions being compared use identical splits. A class whose labelled pixels do not admit a cut leaving at least fifteen pixels on each side after the buffer is removed would be dropped, although no such case occurred in the ten scenes used here.

### 2.7. Controlled Dimensionality Experiments

The experiments in this subsection were designed to separate the effect of ambient dimensionality from the effect of uninformative spectral variance. Three sweeps were used: the first two set dimensionality against noise by increasing the number of bands by an equal amount, while the third varies where the variance is placed in the scene by corrupting the existing bands without changing the number of bands. In the noise sweep, *mD* independent Gaussian noise bands are added to *D* real bands, with the multiplier m={0, 0.5, 1, 2, 4, 8}. In the redundancy control sweep, the same number of bands is added, but each new band is formed by adding a small perturbation to a random linear combination of the real bands. Ambient dimensionality is thereby increased while the genuine class information is preserved. The loss in the redundancy sweep isolates the pure effect of dimensionality, whereas the additional loss in the noise sweep quantifies the contribution of noise. The noise realizations were generated deterministically, so that all classifiers see the same corrupted data at every level. The third sweep corresponds to the way real sensor degradation forms, and six degradation structures were used. In homoscedastic degradation, an independent Gaussian field of equal variance was added to every band. In band-dependent degradation, the per-band noise standard deviation was set by Equation (9); here, $SNR_{b}$ was distributed at equal intervals between fifteen and forty-five decibels and then shuffled at random across bands, so that the noise level varies from band to band without varying monotonically with wavelength. The two added-band sweeps differ in what the added bands contain, and this distinction is the point of the comparison. In the noise sweep, each added band is pure noise: an independent Gaussian field with no relation to the scene, carrying no class information. In the redundancy control sweep, each added band is a noisy signal band, a random linear combination of the real bands with a small perturbation, so that it carries the same class information as the original data in a new direction. Both sweeps raise the ambient dimensionality by exactly the same amount, so any difference in accuracy between them is attributable to the information content of the added bands rather than to their number:

(9) $$\sigma_{b}=10^{-{SNR}_{b}/20}$$

In spectrally correlated degradation, the noise was drawn from a first-order autoregressive covariance along the spectral axis. In Equation (10), $g_{i}$ denotes an independent standard Gaussian vector, L the Cholesky factor of the covariance, and $n_{İ}$ the resulting correlated noise vector:

(10) $$\Sigma_{{bb}^{\prime }}=\rho^{\left|b-b^{\prime }\right|},\;\rho =0.9,\;\Sigma =LL^{⊤},\;n_{i}=Lg_{i}$$

In striping, fifteen per cent of the columns were selected independently in each band and a constant offset drawn from the standard normal distribution was added to each selected column, so that the stripes are not shared across bands. In deadlines, three per cent of the columns were selected independently in each band and replaced by the band mean; the added difference is therefore not noise but removed signal. The mixed structure is a weighted combination of the first four structures. In Equation (11), each component is shown normalized to unit variance, and, because the square roots of the weights are used, the four components contribute to the total added variance in the proportions w:

(11) $$N_{mix}=\sum_{k=1}^{4}\sqrt{w_{k}}\;{\tilde{N}}_{k},\;w=\left(0.4,\;0.3,\;0.2,\;0.1\right)$$

Finally, each structure was rescaled so that the ratio of added variance to signal variance equals the target η in Equation (12), and the structures were evaluated at η=0.5 and η=2.0. The realised ratio was verified numerically in every condition, so that the equal-variance constraint was applied exactly rather than approximately. Because the severity of these conditions cannot be compared directly with the multiplier of the added-band sweep, the two designs were compared at matched severity by interpolating the added-band curve at the severity value reached in each in-band condition:

(12) $$\eta =\frac{Var\left(N\right)}{Var\left(X\right)}$$

A nonlinear redundancy control was added in order to determine whether the harmlessness of the added bands is an artefact of their being constructed within the linear space of the original data. This control repeats the redundancy sweep on the three calibration scenes at two injection levels, m=1 and m=4, with the same classifiers, the same leakage-aware splits, and ten repetitions. The number of added bands is the same as in the linear sweep and equals mD. The two conditions differ only in how the bands are generated. In the linear condition, each additional band was formed by adding a small perturbation, of one-twentieth of the standard deviation, to a random Gaussian combination of the real bands. In the nonlinear condition, three transforms were applied in equal proportions and cyclically along the added bands: the square of the projection onto a random unit vector, the product of two randomly chosen bands, and the hyperbolic tangent of one and a half times the projection onto a random unit vector. Each additional band was centered and scaled to unit variance in both conditions, so that differences of scale were eliminated. The degree to which the added bands remain within the linear space was moreover not assumed but measured: for twenty-five randomly chosen additional bands, each band was predicted from the original bands by ordinary-least-squares regression on a random subsample of four thousand pixels, and the mean coefficient of determination is reported. The three transforms were not chosen arbitrarily but to span the elementary ways in which a band can leave the linear span while remaining a deterministic function of the data: a second-order term, an interaction between two bands, and a saturating monotone map. Together they cover quadratic, multiplicative, and bounded nonlinearity, which are the forms most commonly used to construct nonlinear features in spectral analysis. The measured coefficient of determination confirms that they achieve the intended effect, falling from 0.994 in the linear condition to 0.757 in the nonlinear one.

The training protocol under degradation is identical in all conditions and is stated explicitly here to avoid ambiguity. The degradation was applied to the entire scene before splitting, so that the training and test pixels carry the same degradation. The classifiers were furthermore retrained from scratch for every combination of scene, structure, and severity. Likewise, all data-dependent steps, including per-band scaling and the principal component transform of the convolutional front end, were estimated on the corrupted training pixels of the relevant split and applied unchanged to the corrupted test pixels. The experiment therefore measures not the transfer of a model trained on clean data to corrupted data, but the situation encountered when a single acquisition has to be mapped, that is, the extent to which a classifier can adapt within a corrupted scene.

In order to test whether the findings depend on an idealized noise model, the same sweep was also repeated on the Indian Pines, Pavia University, and Salinas scenes with three structured noise types in addition to independent Gaussian noise: noise correlated along the band axis, column-wise striping, and band-dependent signal-to-noise ratio. These three types correspond to degradation sources reported for real hyperspectral sensors, in which shot noise makes the noise level band-dependent and atmospheric effects behave coherently across bands; the noise is therefore neither spectrally white nor spatially unstructured [39]. For the comparison to be fair, the same number of bands and the same total variance were added in all four types, and only the structure of the noise was changed.

### 2.8. Severity-Based Regression Rule and Statistical Analysis

The regime-based rule in Section 2.3 selects the classifier according to the structure of the degradation. As a benchmark for that rule, a second rule that uses only the magnitude of the degradation was defined. Pooling all operating points, a linear regression of overall accuracy on the severity index was fitted for each classifier c, and these models were used as in Equation (13) to select the classifier with the highest predicted accuracy at a given severity level:

(13) $${\hat{OA}}_{c}=\alpha_{c}+\beta_{c}L_{conc},\;c^{*}=arg\underset{c}{max}({\hat{OA}}_{c})$$

The performance of the rule is reported through the regret defined in Equation (14), which measures how far the selected classifier falls behind the best classifier at that operating point:

(14) $$R=\underset{c}{max}\left(OA_{c}-OA_{c^{*}}\right)$$

The rule was evaluated out of sample by leaving one dataset out entirely at a time; the regret is reported separately for the undisturbed and the noise-injected regimes. This separation is necessary because the five synthetic noise levels dominate the single undisturbed level numerically, so that a pooled mean alone would be misleading. The monotone relationship between severity and accuracy was, moreover, assessed with the Spearman rank correlation coefficient, which was preferred because it requires no assumption of a linear relationship and the overall accuracy distributions are not normal. Since pooled operating points from the same scene are not independent, this dependence was addressed in three complementary ways. First, within-dataset slopes were computed separately for each scene and pooled by treating the datasets as independent units; their difference from zero was tested with a one-sample t test. Second, a linear mixed-effects model with a random intercept, and a random slope where it converged, was fitted for each classifier with the dataset as the grouping factor. Third, a dataset-level cluster bootstrap that resamples all scenes and makes no distributional assumption provided confidence intervals for the slope. Where the mixed model and the bootstrap disagreed, the bootstrap result is reported. The same slopes were also computed separately in the realistic (m≤1) and extreme (m≥2) regimes in order to confirm that extreme injection levels do not drive the result. For the comparison of classifiers, the Friedman test and the Nemenyi post hoc procedure were used, these being the standard methods for multiple comparisons across several datasets [54]. The datasets were taken as blocks (N=10) and each block value was computed as the mean of the relevant scene over ten seeds. This block definition was preferred because treating repeated splits of the same scene as independent blocks would amount to pseudoreplication. This test was applied both to undisturbed accuracy and to the noise-induced accuracy loss, so that the ranking was assessed not only for clean performance but also for robustness. Finally, the superiority of the proposed selection rule over fixed policies was tested with the paired Wilcoxon signed-rank test, and multiple comparisons were corrected with the Holm procedure. Although the values shown in the tables are rounded, derived quantities and statistical tests were computed from full-precision results; recomputation from the tabulated values may, therefore, differ at the level of 0.001.

### 2.9. Map-Level Demonstration

In order to express the diagnosis in terms of map quality rather than aggregate accuracy alone, three scenes, namely, Salinas, Pavia University, and Indian Pines, were corrupted in band with two opposing degradations at a variance ratio of two: spatially local striping and spectrally correlated noise spread across the entire spectrum. For each degradation, the selected and avoided classifiers were not chosen by hand but read from the mean accuracy losses obtained in the in-band degradation experiments, and the family with the lower loss was selected. Both classifiers were then trained with the same spatially disjoint training pixels used throughout the study and applied to every pixel of the scene to obtain a full thematic map. Overall accuracy, average accuracy, and Kappa, in contrast, were computed on the test pixels only. The spatial structure of the errors was summarized with an excess clustering statistic, defined as the proportion of erroneous test pixels lying in connected error blocks of at least fifty pixels minus the proportion expected when the same number of errors is placed at random among the test pixels; the expectation was averaged over twenty random placements. This correction is necessary because, at high error rates, isolated errors may merge into large connected components by chance alone. A value close to zero therefore indicates pixel-level errors, whereas a large positive value indicates field-scale errors. Finally, the convolutional network used in this demonstration is an equivalent but simplified version of the network used elsewhere in the study and was trained on a single split, since the experiment is explanatory rather than inferential. The variance ratio of two was chosen as a representative high-severity condition from the two levels used throughout the in-band experiments rather than as a physically calibrated degradation level. It corresponds to added variance twice the signal variance, which is severe by the standards of real sensor degradation; it is used here because the purpose of the demonstration is to make the consequence of the regime distinction visible on a map, and the lower level of 0.5 produces differences too small to be seen at map scale.

## 3. Results

The results are presented in four stages. Section 3.1 establishes the precondition of the diagnosis; after reporting the intrinsic dimensionality of the scenes and the label-free diagnostic measures, it separates the effects of dimensionality and noise on accuracy at an equal number of bands and compares the behavior of the classifier families under degradation. Section 3.2 evaluates the label-free indicators, addressing, in turn, the predictive power of the severity index, the effect of the in-band degradation structures, the gate and structure classification performance of the two-layer rule, and the validation obtained on real sensor degradation. Section 3.3 examines the mechanism underlying the observed robustness difference and demonstrates the decision value of the diagnosis for classifier selection at the map level. Section 3.4 reports the extent to which the evaluation protocol and the architectural choices affect the results. Unless stated otherwise, all accuracy values were obtained under the leakage-aware spatial split protocol defined in Section 2.6.

### 3.1. Dimensionality, Noise, and Fragility

The estimated intrinsic dimensionality of each scene lies between 3.76 and 8.55 and is an order of magnitude below the nominal number of bands; the redundancy measure lies between 0.920 and 0.969. The complementary diagnostic measures point in the same direction: relative contrast varies between 2.52 and 11.88 and hubness skewness between 0.02 and 1.24; the highest hubness values occur in Loukia and Indian Pines and the highest severity values in Indian Pines and Salinas. All values are given in Table 2.

**Figure 2:**
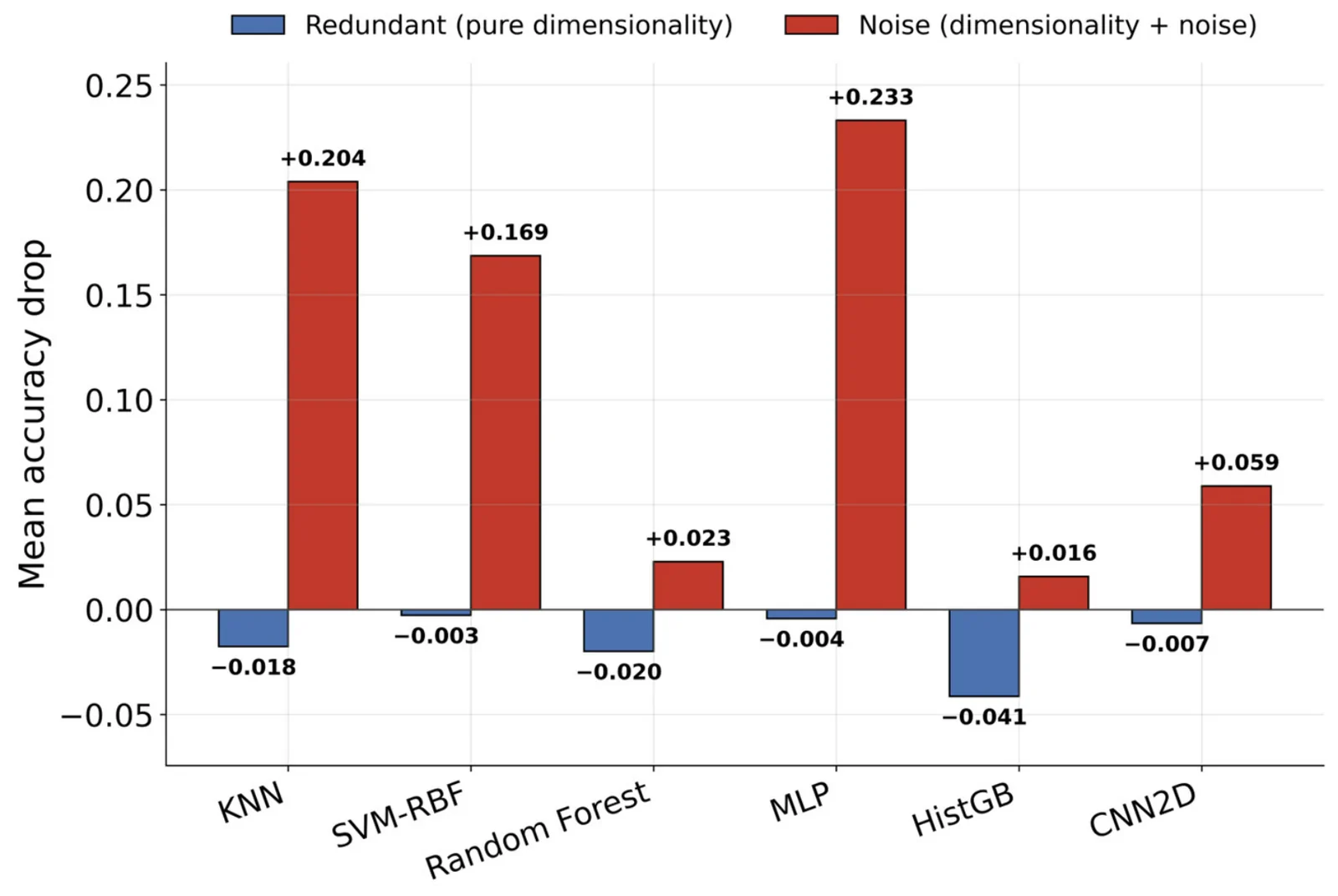
Accuracy loss under redundancy and noise injection.

The most apparent pattern in Table 2 is that, despite the variation in the sensor and number of classes, the intrinsic dimensionality remains well below the nominal number of bands in all scenes. This regularity shows that a low effective dimensionality is not a coincidence specific to a single scene but a shared property of the hyperspectral data examined here. Most of the spectral variation, therefore, lies in a low-dimensional subspace, and whether nominal dimensionality alone harms accuracy can be tested directly. Since the natural severity values of the undisturbed scenes are, moreover, concentrated in a narrow range, higher severity levels can be reached only through the controlled noise sweep. The outcome of this test is presented in Figure 2, where the redundancy and noise sweeps are compared.

**Figure 3:**
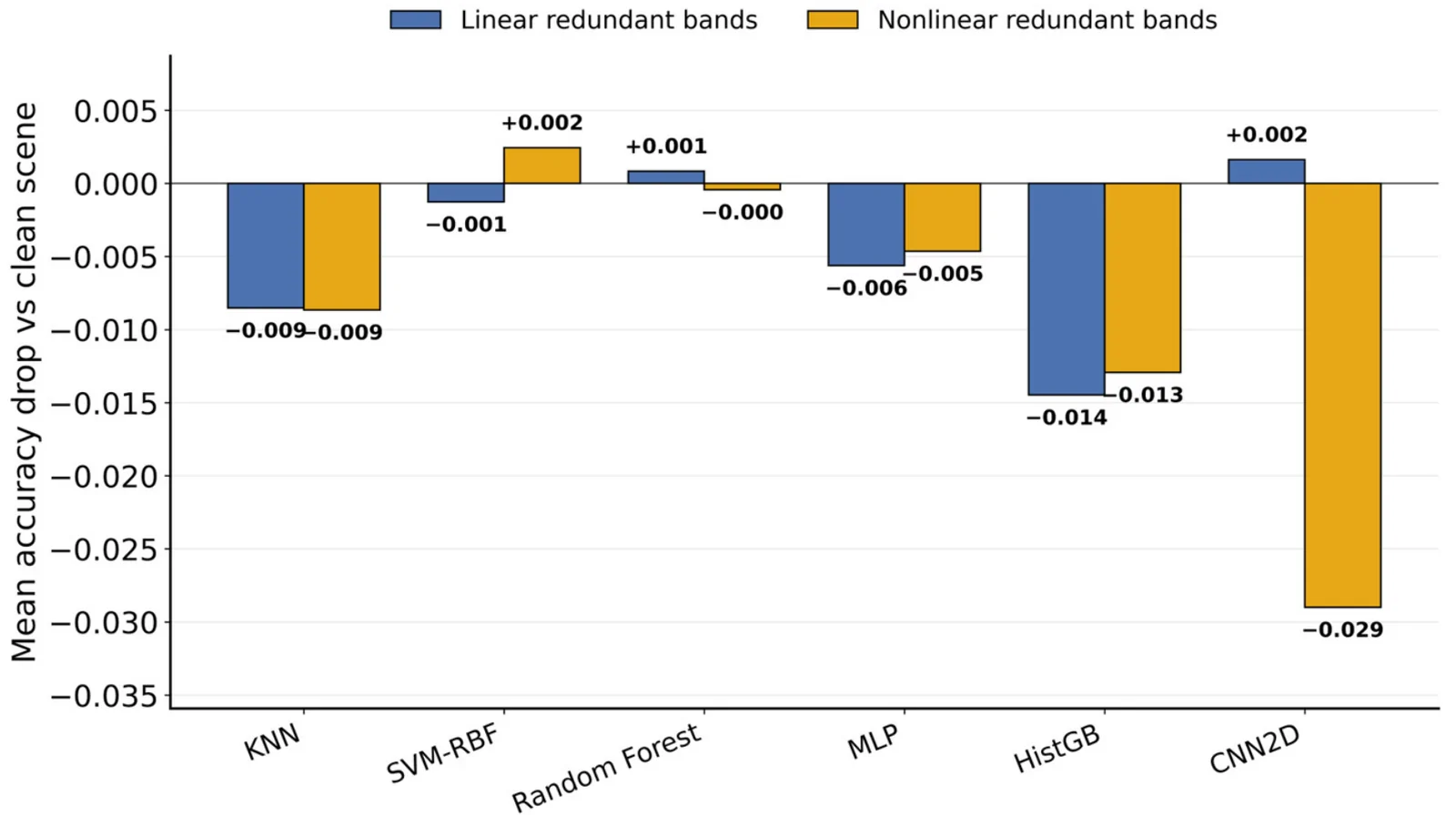
Accuracy difference for linear and nonlinear redundant bands.

Figure 2 compares the two controlled sweeps. Adding informative but redundant bands changed accuracy hardly at all for any classifier: the mean loss over the ten datasets is −0.018 for KNN, −0.003 for SVM–RBF, −0.004 for MLP, and −0.007 for CNN2D; negative values were also obtained for the two tree ensembles, with −0.020 recorded for the random forest and −0.041 for HistGB. That all values are close to zero and most are slightly negative accordingly shows that adding informative bands does not reduce the accuracy and may even raise it very slightly. When the same number of independent noise bands is added, in contrast, the accuracy of the distance-, kernel-, and fully-connected-network-based methods drops markedly. The mean losses are 0.204 for KNN, 0.169 for SVM–RBF, and 0.233 for MLP, whereas the ensembles and the convolutional network remained robust with limited losses of between 0.016 and 0.059. Since the redundant bands raise ambient dimensionality by exactly the same amount as the noise bands, yet do not lower accuracy, the degradation observed in the noise sweep can indeed be attributed to noise rather than to dimensionality. What is frequently measured as a dimensionality effect in the literature is, in these experiments, the effect of uninformative spectral variance. A numerical summary of the losses is given in Table 3. These redundant bands, however, are random linear combinations of the real bands and, therefore, remain within the linear space of the original data. A method able to represent this space might be expected to be unaffected by such bands for purely algebraic reasons. To test this possibility, the same number of bands was added on the three calibration scenes at injection levels of one and four, this time through nonlinear transforms: the square of a random projection, the product of two bands, and a saturating transform of a random projection. Because these bands are deterministic functions of the original data, they carry no new information and remain on the same low-dimensional manifold, but they are no longer within the linear space. This was not assumed but verified: the mean coefficient of determination for the linear predictability of the added bands from the original bands was 0.994 in the range of 0.990–0.996 in the linear condition, and fell to 0.757 in the range of 0.514–0.850 in the nonlinear condition. The lowest values occurred in Pavia University. The outcome of the control experiment is shown in Figure 3.

Accuracy remained largely unchanged: the mean difference relative to the clean scene varied between −0.014 and +0.002 for five of the six classifiers and reached −0.029 only for the convolutional network in the nonlinear condition, a value of the same order of magnitude as those obtained under linear redundancy. The harmless effect of additional dimensionality is, therefore, not an artefact of the added bands being constructed within the linear space. The single value that departs from the others, the loss of 0.029 for the convolutional network in the nonlinear condition, follows from the principal component front end being a linear projection. Bands constructed by a nonlinear transform do not lie in the span of the original bands, so the twenty retained components represent them less completely than they represent the linearly redundant bands, and a small part of the variance the network receives is therefore unmodelled. The magnitude remains of the same order as the values obtained under linear redundancy, so it does not affect the conclusion; it is reported here because it is the one systematic difference between the two conditions.

Table 3 states numerically the visual distinction seen in Figure 2. That the redundancy loss column is close to zero or negative for every classifier confirms quantitatively that an increase in dimensionality alone is harmless. The noise loss column, in contrast, separates the methods into two groups, while the noise contribution column isolates the part of the loss that arises from noise alone. Read together, the columns show that noise sensitivity increases regularly towards MLP, KNN, and SVM–RBF across all datasets, while remaining limited for the tree ensembles and the convolutional network.

Bands were added in both experiments so far. Real sensor degradation, however, does not add bands but forms on top of the existing ones. In the third experiment, the number of bands was therefore held fixed, the existing bands were corrupted, and the two designs were compared at a matched severity by aligning them on the severity index. Averaged over the ten datasets, in-band degradation is more harmful than added bands of the same severity for every classifier: the mean difference in overall accuracy is 0.110 for SVM–RBF, 0.103 for HistGB, 0.101 for the random forest, 0.098 for MLP, 0.089 for KNN, and only 0.009 for the convolutional network, which already removes the spread noise through its principal component front end. That the per-classifier mean is positive for every family thus shows that the effective quantity is not only the amount of uninformative variance but where that variance is placed. At the level of individual cells, the difference is not always positive: it is slightly negative for the band-dependent structure in three of the six classifiers, and, for the convolutional network, it is negative under the homoscedastic, band-dependent, correlated, and mixed structures. These exceptions are consistent with the principal component front end removing noise spread across the existing bands as effectively as noise in added bands. Of the 720 matched points, 714 lie within the interpolation range of the added-band sweep. All six exceptions are the dead-line condition in a single scene, where the removal of the signal lowered the severity below the range of the sweep, and they are reported separately. The comparison is summarized in Table 4. The comparison was tested with the paired Wilcoxon signed-rank test over the 119 matched scene-structure points available for each classifier, with the Holm correction across the six classifiers. In-band degradation is significantly more harmful for the five classifiers that do not remove spread noise through a front end, with Holm-corrected p values between $8.2\times 10^{-14}$ and $2.5\times 10^{-18}$. For the convolutional network. the difference of 0.009 is not significant (Holm-corrected p=0.64), which is consistent with its principal component front end removing spread noise as effectively in band as in added bands.

In Table 4, a positive value means that in-band degradation is more harmful than added bands at matched severity. The largest differences occur in the correlated degradation column, reaching +0.261 for the random forest, +0.256 for HistGB, and +0.245 for SVM–RBF. In the band-dependent column, the values remain between −0.031 and +0.019, that is, close to zero. The convolutional network row is negative for four of the six structures and takes positive values only in the striping and dead-line columns.

**Figure 4:**
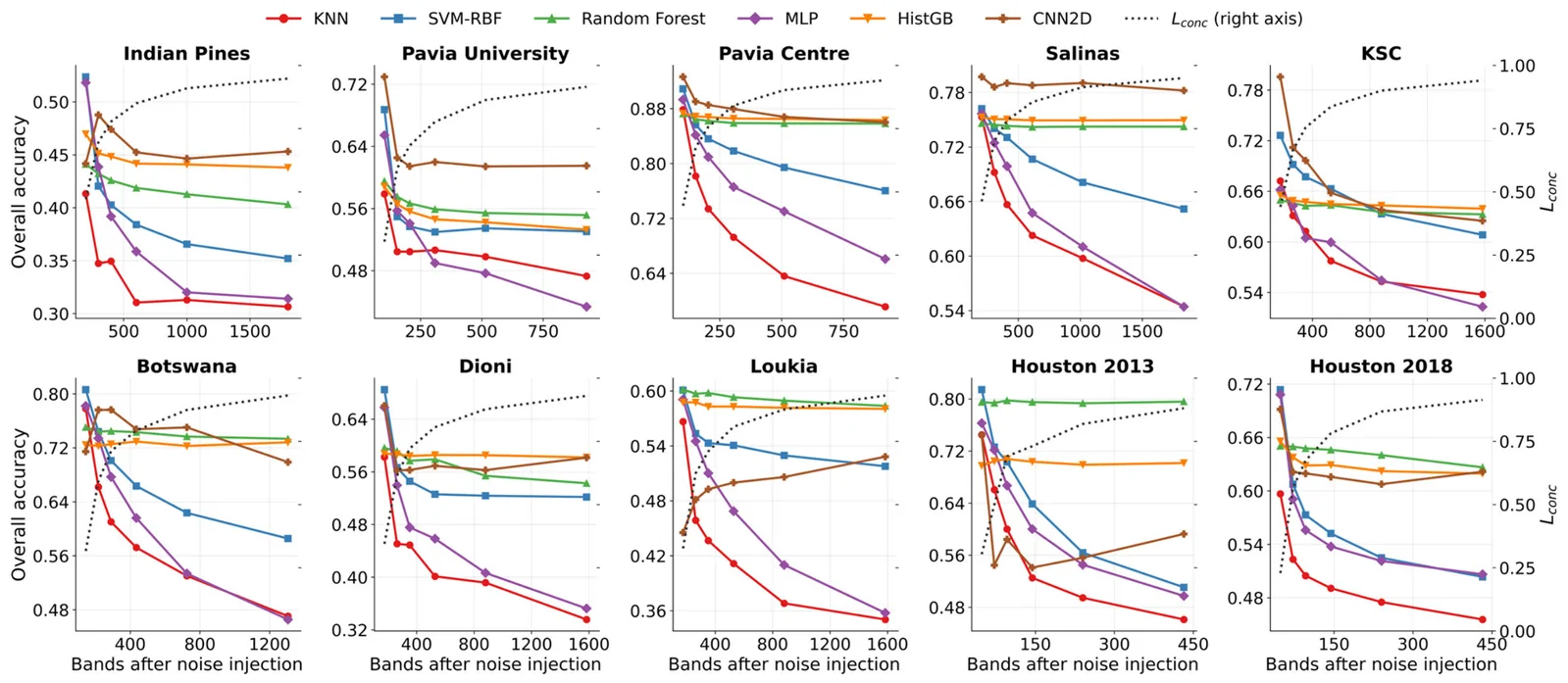
Accuracy under progressive noise band injection, by dataset.

That the tree ensembles and the convolutional network appear robust in every condition of the added-band sweep is only part of the story. The matched-severity comparison above has already shown that in-band degradation is more harmful; below, the robustness of these methods is shown to depend on the structure of the noise. Now that noise has been established as the decisive factor, how classifier behavior differs under noise can be examined. To this end, the noise sweep across all ten scenes is presented in Figure 4.

**Figure 5:**
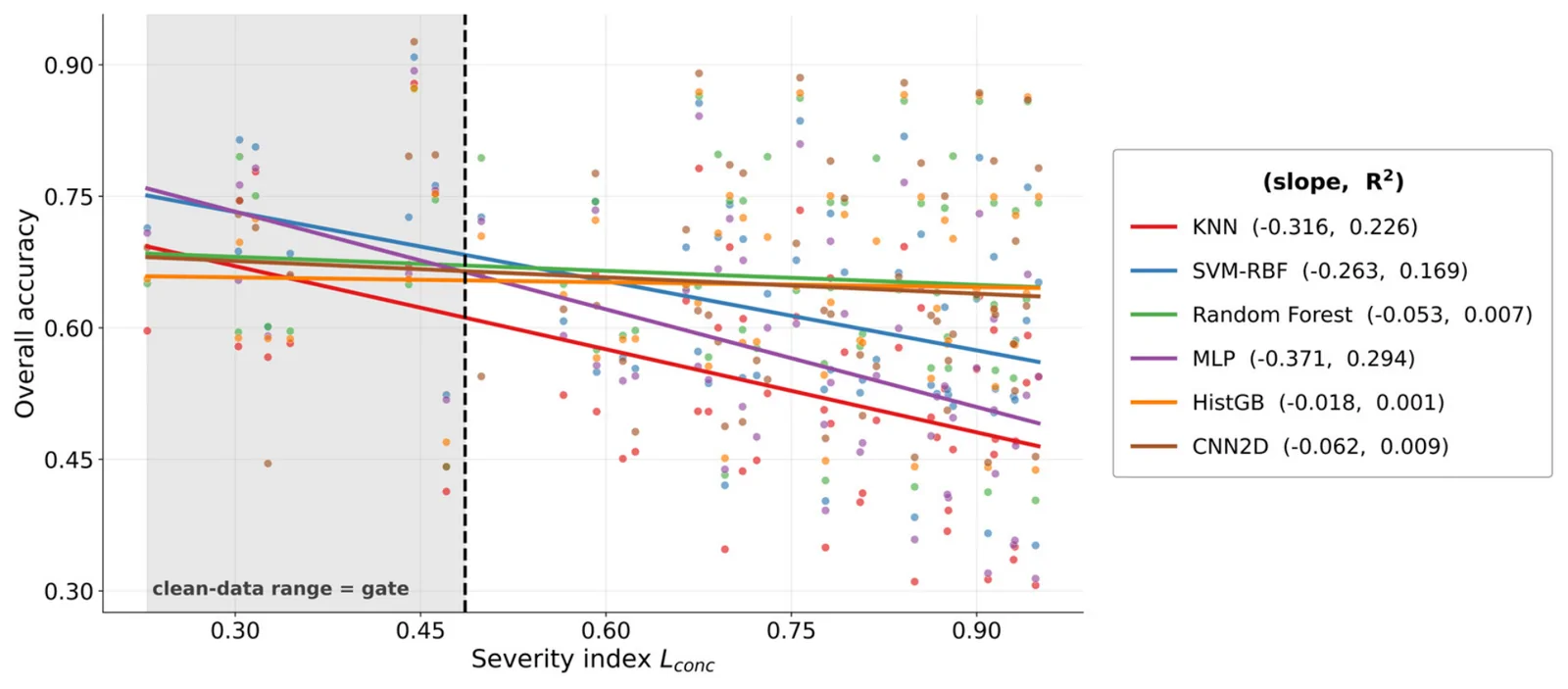
Accuracy versus the severity index over pooled operating points.

Figure 4 carries the summary finding of Figure 2 to ten individual scenes and thereby demonstrates its generalizability. The same pattern recurs in all ten panels: as noise bands accumulate, the distance-, kernel-, and fully-connected-network-based curves fall rapidly, whereas the curves of the tree ensembles and the convolutional network remain almost flat. This consistency therefore shows that the distinction is not specific to a single dataset or sensor and holds across scenes with different numbers of classes and bands. The monotone rise in the dotted severity curve with noise in each panel moreover makes visible that the index tracks the noise injection directly and without labels, and foreshadows the indicator–accuracy relationship quantified below. Finally, the starting point of the curves, that is, the undisturbed accuracy in the noise-free condition, is independent of the slope of the decline; a method that starts high on clean data does not thereby become robust to noise. Although the curves of the robust methods fluctuate slightly at the highest noise level in some scenes, these movements are negligible beside the systematic decline of the fragile methods. In each panel, the dotted curve shows the severity index read from the right-hand axis. When the operating points of all scenes are pooled on a single axis, the relationship between the index and accuracy is seen in Figure 5.

Figure 5 summarizes the relationship between the index and accuracy quantitatively by pooling all noise levels of the ten scenes on a single axis. The shaded band shows the range spanned by the undisturbed scenes, and its upper bound the severity gate. The slope of the line fitted to each classifier is, accordingly, a direct measure of that method’s fragility with respect to severity: the KNN, SVM–RBF, and MLP lines slope markedly downwards, whereas the random forest, HistGB, and convolutional network lines are almost flat. The scene-wise trend of Figure 4 is thereby reduced to a single pooled summary of fragility. The dashed vertical line marks the highest severity value observed among the undisturbed calibration scenes and, thus, defines the severity gate. The entire region to the right of the threshold corresponds to the higher severity values reached through noise injection and shows how a classifier would behave there in practice. The width of the point cloud is expected, since the pooled points come from different scenes and accuracy is affected by many factors besides severity.

**Figure 6:**
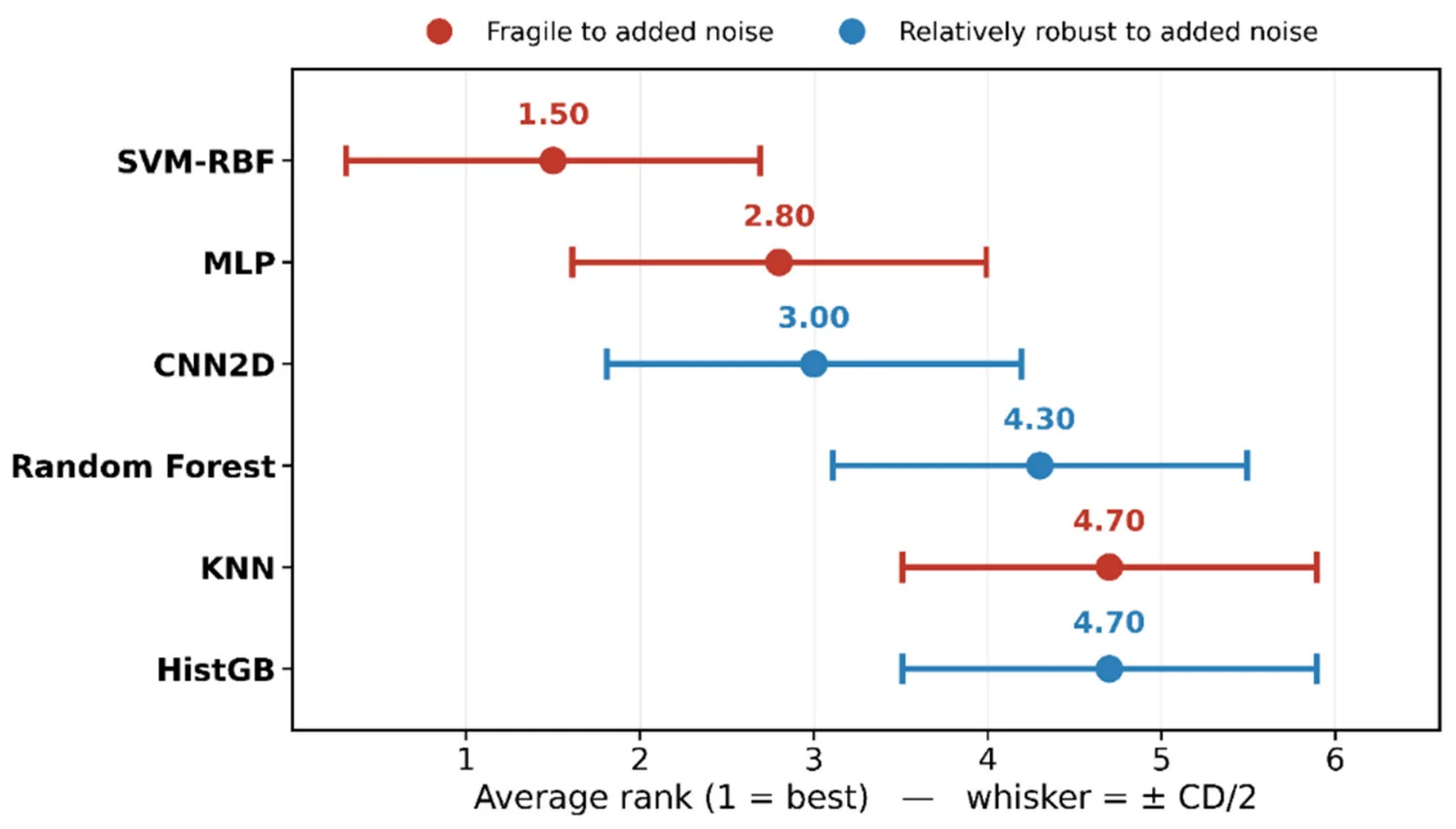
Classifier ranking on undisturbed scenes.

The information carried by the figure is, therefore, not the position of individual points but the difference in the slope that separates the fragile from the robust methods. The slopes of the fitted lines are −0.32 for KNN, −0.26 for SVM–RBF, and −0.37 for MLP, against −0.05 for the random forest, −0.02 for HistGB, and −0.06 for the convolutional network. Within-dataset slopes computed by treating the datasets as independent units sharpen the distinction further: −0.398 for MLP, −0.350 for KNN, and −0.285 for SVM–RBF; −0.038 for the random forest, −0.027 for HistGB, and −0.104 for CNN2D. The slopes are smaller for the robust methods; for the random forest and HistGB, they remain at roughly one-tenth of those of the fragile methods, while, for the convolutional network, the difference is more limited. Whether these slopes are significant at the dataset level is assessed with the mixed effects model and cluster bootstrap in Section 3.2.1. That the ranking differs on undisturbed data is seen in Figure 6. The lines in Figure 5 are ordinary-least-squares fits of overall accuracy on the severity index, computed over the sixty pooled operating points. Their goodness of fit follows the same division as the slopes: the coefficient of determination is 0.294 for MLP, 0.226 for KNN, and 0.169 for SVM-RBF, with p below 0.01 in all three, against 0.009 for the convolutional network, 0.007 for the random forest, and 0.001 for HistGB, none of which is significant. The values are modest even for the fragile methods, since the pooled points come from scenes that differ in many respects besides severity; the quantity the figure conveys is the difference in the slope rather than the proportion of the variance explained.

Figure 6 orders the classifiers by their mean Friedman rank on undisturbed data. The marker is the mean of the ten per-scene ranks, not a median or a quantile, and the horizontal bar is not a quantile interval: it extends half the Nemenyi critical difference to either side of the marker, the critical difference being 2.38 for six classifiers and ten scenes at the five per cent level. The bar is, therefore, a fixed-width significance interval of the same length for every classifier, which is why the markers lie at its center by construction. Methods whose bars overlap cannot be distinguished statistically. The lowest rank, that is, the best undisturbed performance, belongs to SVM–RBF, marked in red; the red color, however, indicates that this method is fragile to noise. The random forest and HistGB, which are comparatively robust to added independent noise, are, by contrast, placed lower on the undisturbed data. That the color code and the rank axis are independent of each other confirms, at the statistical level, the conclusion that undisturbed accuracy and noise robustness are distinct properties. In terms of significance, the figure separates the upper methods from the lower ones; because the critical difference computed with ten datasets is comparatively wide, however, small differences between adjacent ranks are not significant. The cautious conclusion to be drawn from the figure is therefore not a single definitive ordering but a separation of the methods into an upper and a lower group. The mean ranks read from the figure are 1.50 for SVM–RBF, 2.80 for MLP, 3.00 for the convolutional network, 4.30 for the random forest, and 4.70 for both KNN and HistGB. The Friedman test with the datasets as blocks rejected the equality of the classifiers, with a chi-square value of 23.60 and a *p* value below 0.001. The scene-wise accuracies on which the ranking rests are given in Table 5.

According to the last row of Table 5, the highest mean overall accuracy on undisturbed data is reached by SVM–RBF with 0.723, followed by MLP with 0.699, the convolutional network with 0.695, the random forest with 0.670, HistGB with 0.659, and KNN with 0.656. Two points stand out when the table is read. First, no classifier is superior across all datasets on undisturbed data. Although SVM–RBF leads on average, the convolutional network gives the highest accuracy on the Pavia University, Pavia Centre, Salinas, and Kennedy Space Center scenes; the best method therefore depends on the scene. Second, the standard deviations are limited in most cells; the convolutional network, however, is markedly more variable in some scenes and reaches 0.181 in Pavia University. The significance of the differences between the methods was therefore based not on the standard deviations alone but on the Friedman and Nemenyi results reported below. That the undisturbed ranking in this table does not coincide with the noise robustness ranking reported above supports, once more, the finding that the two properties are independent.

### 3.2. Label-Free Degradation Indicators

#### 3.2.1. Predictive Power of the Severity Index

Whether this robustness difference between classifiers can be predicted quantitatively by the proposed severity index is assessed below. Over the pooled operating points, the severity index is significantly and negatively associated with accuracy for KNN, SVM–RBF, and MLP, whereas the association is not significant for the random forest, HistGB, and the convolutional network; the *p* value is 0.001 or below for the three fragile methods, and the Spearman coefficients are given in Table 6. This result is internally consistent, because the robust methods are largely unaffected by noise and their accuracy therefore does not track the index.

Generalization to new scenes was assessed with leave-one-dataset-out validation, in which an entire scene is left out at a time. This procedure was preferred over the leave-one-point-out approach used in some earlier analyses, because removing only a single operating point leaves the other points of the same scene in the training set and thereby reintroduces into the validation procedure the very spatial dependence that is avoided throughout the rest of the study. The correlation coefficients, the slopes, and the coefficients of determination under this protocol are given in Table 6.

Table 6 separates the six classifiers into two statistically distinct groups. For the fragile methods, the Spearman coefficient is significant and negative and the slope magnitude is pronounced; for the robust methods, the coefficient is not significant and the slope is close to zero. Out of sample, in contrast, the picture is weaker and is not uniform even within the fragile group: the leave-one-dataset-out coefficient of determination is positive but small for MLP and KNN, at 0.125 and 0.040, respectively, and negative for SVM–RBF at −0.039. For the three robust methods, it is clearly negative: −0.244 for the random forest, −0.249 for HistGB, and −0.225 for the convolutional network. A negative value means that the fitted line predicts a held-out scene worse than that scene’s own mean. This is to be expected for robust methods whose accuracy scarcely varies with severity. The index is, therefore, not a model that predicts accuracy across scenes but a diagnostic variable that flags increasing degradation, and it does so most reliably for the fragile families. This shows that the index carries no predictive value for the robust methods and is a diagnosis directed specifically at the fragile families.

Whether the relationship arises only from extreme injection levels was further tested. When the operating points are divided into the realistic (m<=1) and extreme (m>=2) regimes, the within-dataset slopes remain significant and of similar magnitude in the realistic regime as well: −0.310 for KNN, −0.271 for SVM–RBF, and −0.281 for MLP, with a *p* value below 0.001 in all three; −0.025 was obtained for the random forest and −0.026 for HistGB. The roughly eleven-fold difference between the fragile and the robust methods is preserved in this regime, so the finding is not a by-product of extreme conditions. In the extreme regime, the slopes steepen further, reaching −0.738 for MLP and −0.484 for KNN, with the pooled values lying between the two regimes. The convolutional network falls outside this pattern: its slope of −0.126 in the realistic regime drops to −0.026 in the extreme regime—that is, the degradation saturates as noise increases. This behavior is consistent with the front-end variance analysis reported in Section 3.3; at high injection levels, the principal component front end removes almost all of the noise variance, so that additional noise has no further effect. It should be noted that the Spearman coefficients are not compared directly across regimes, because the narrowing of the severity range lowers the correlation mechanically. The comparison across regimes was, therefore, made through the slopes. Since the operating points from the same scene are not independent, the slope was re-estimated with two methods that account for this dependence, and the results are reported in Table 7.

Table 7 places the results of the two methods side by side. The linear mixed-effects model, with the dataset as a random effect and a random slope that converged in every case, gave fixed-effect slopes of −0.350 for KNN, −0.286 for SVM–RBF, and −0.396 for MLP, with p<0.001 in all cases. Values of −0.038 for the random forest, −0.027 for HistGB, and −0.103 for the convolutional network were obtained in contrast. Accordingly, the dataset-level cluster bootstrap, which resamples all scenes and makes no distributional assumption, gave the same result for the fragile group but behaved more conservatively for the robust group: the confidence intervals of the random forest, HistGB, and the convolutional network all include zero, with two-sided *p* values of 0.254, 0.665, and 0.193, respectively. The confidence intervals of the three fragile methods, by contrast, lie entirely below zero. Since the bootstrap result is reported where the two methods disagree, it is accepted that the robust methods show no established severity–accuracy relationship rather than a small but significant one. When the Friedman test is applied to the noise-induced accuracy loss instead of clean accuracy, a chi-square of 36.97 and $p=6.1\times 10^{-7}$ were obtained for the ten scenes, with a Nemenyi critical difference of 2.38. The mean ranks are 1.4 for HistGB, 2.1 for the random forest, and 2.9 for the convolutional network; and 4.5 for SVM–RBF, 4.6 for KNN, and 5.5 for MLP. The robustness ranking is thereby confirmed statistically in its own right and shown not to be a by-product of clean accuracy. The out-of-sample prediction errors of the severity index are given in Table 8.

Table 8 summarizes the practical accuracy of the index. The coefficients of determination remained low even for the fragile methods, whereas the prediction error was similar and moderate across the two groups: the mean absolute error of the predicted overall accuracy lies between 0.100 and 0.119 for all six classifiers. The index is, therefore, presented not as a precise predictor of accuracy but as a variable that flags noise sensitivity before classification.

#### 3.2.2. Effect of the In-Band Degradation Structures

**Figure 7:**
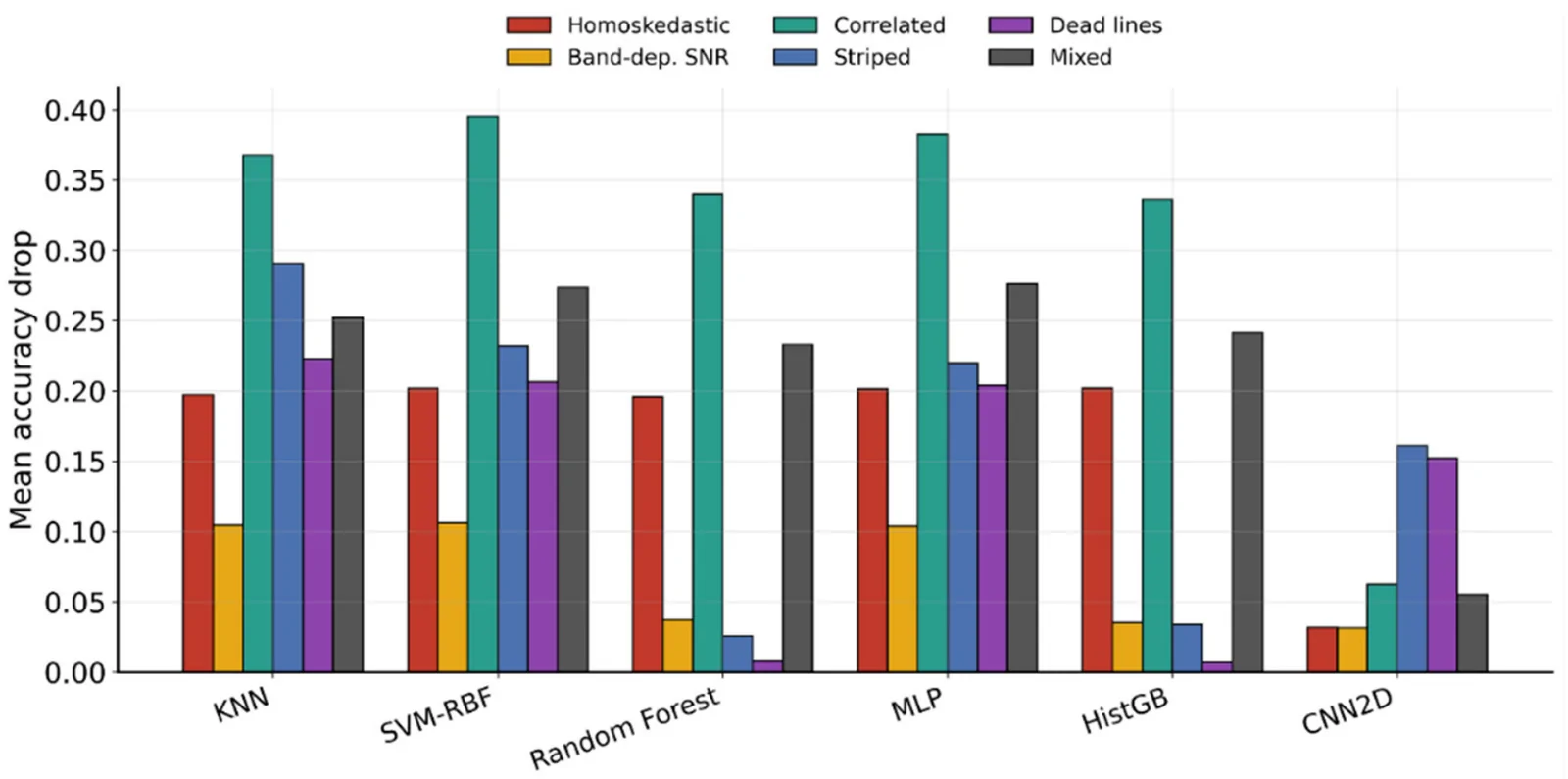
Accuracy loss under six in-band degradation structures (η=2).

The added-band experiment holds the noise structure fixed as independent Gaussian bands. Real degradation, however, is neither entirely independent nor confined to separate bands. The in-band experiment of Section 2.7, therefore, corrupts the existing bands of the ten scenes with six structures at two severity levels: homoscedastic, band-dependent, spectrally correlated, striping, deadlines, and a realistic mixture. The mean accuracy loss at a high severity is given in Table 9 and shown by family and structure in Figure 7.

Table 9 places the mean accuracy losses of the six classifiers under the six degradation structures side by side. The clearest distinction between the rows is that the tree ensembles and the convolutional network behave in opposite ways across the columns: the tree ensembles show high loss in the correlated and homoscedastic columns, and low loss in the striping and dead-line columns, whereas the reverse holds for the convolutional network. For the three fragile methods, the loss is high in all columns.

The resulting pattern reverses the uniform robustness suggested by the added-band view. The two-regime structure is, in fact, already present at low severity and sharpens as the severity increases. Treating the homoscedastic and spectrally correlated structures as spread and striping and deadlines as spatially local, the difference between these two groups for the two tree ensembles rises from 0.136 in the low-severity condition (η=0.5) which is not tabulated separately, to 0.250 at the high severity of Table 9. For the convolutional network, in contrast, the difference in the opposite direction, that is, local minus spread degradation, rises from 0.065 to 0.110. That the pattern appears at both severities therefore shows it to be a structural property of the classifier families rather than an artefact of extreme injection. The regime difference was likewise tested across scenes rather than read from the means alone. Taking each scene as a paired unit, the difference between spread and spatially local degradation is significant for the tree ensembles (median difference 0.250, Wilcoxon p=0.002 over ten scenes) and significant in the opposite direction for the convolutional network (difference −0.110, p=0.004).

Robustness now depends on where the noise resides. Under degradation spread across the spectrum, the tree ensembles are degraded as much as the fragile methods: at high severity, the loss of the random forest is 0.340 under correlated noise and 0.196 under homoscedastic noise, close to the values of 0.368 and 0.197 for KNN, and HistGB behaves similarly. Under spatially local degradation, in contrast, the same ensembles are almost unaffected; the losses of the random forest under striping and deadlines are 0.026 and 0.008, respectively. The convolutional network shows the opposite profile: with losses in the range of 0.031–0.062, it is the most robust family under spread noise; yet, with losses of 0.161 and 0.152 under striping and deadlines, it is among the most fragile, because the convolution layers read the spatial column structure as the signal. No family is, therefore, robust across all noise structures; the ranking of the families depends on the noise regime. It is precisely for this reason that the diagnosis must report not only the magnitude of the degradation but also its type.

#### 3.2.3. Performance of the Gate and the Structure Indicators

**Figure 8:**
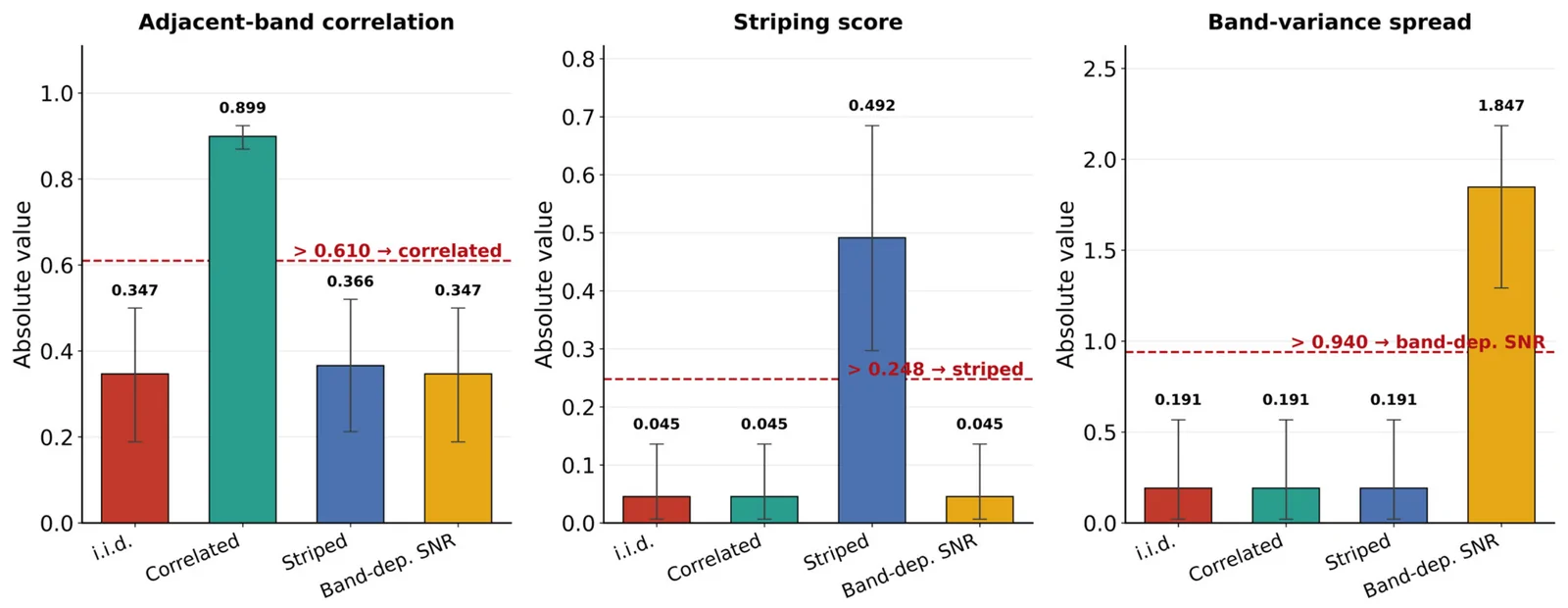
Label-free structure indicators by noise type.

Because classifier selection depends on the structure of the noise, the severity index alone is not sufficient, and the three structure indicators defined in Section 2.2 are evaluated here. The thresholds calibrated with the procedure described in Section 2.3 and their bootstrap confidence intervals are as follows: a threshold of 0.248 with a 95% confidence interval of 0.164–0.331 for the striping score, a threshold of 0.940 with an interval of 0.858–1.016 for the variance spread, and a threshold of 0.610 with an interval of 0.541–0.700 for the adjacent-band correlation. The indicator values by noise type are given in Figure 8, where the dashed lines show these thresholds.

At the gate stage, the severity threshold was set to 0.486, the highest severity value observed in the undisturbed data of the three calibration scenes. Applied unchanged to the seven evaluation scenes, the gate was correct in all 42 conditions, comprising the seven undisturbed scenes and the thirty-five degraded conditions formed by five injection levels in each. Under the more demanding in-band conditions, in contrast, it continued to classify all ten undisturbed scenes correctly and was correct in 106 of the 120 scene–structure combinations; this figure falls to 93/120 when only the 0.539 severity threshold is used and to 103/120 when only the 0.486 severity threshold is used. The higher threshold also misses one of the added-band conditions: the mildest injection level of the Houston 2013 scene, with a severity of 0.499, exceeds the 0.486 threshold but falls below the 0.539 threshold. The band-variance spread was further examined as a third gate criterion but was not used, since it was activated in only one of the 120 conditions and identified no additional case that the other two criteria missed. The adjacent-band correlation, by contrast, cannot be used in the gate: remaining between 0.924 and 0.997 in the undisturbed scenes, it lies far above the 0.610 threshold that defines the correlated noise, and a gate including it would assign all ten undisturbed scenes to the degraded class, yielding zero specificity. The performance of the gate under these configurations is summarized in Table 10.

Table 10 demonstrates the necessity of the two-part structure of the gate. When the severity and striping criteria are used together, 106 of the in-band conditions are classified correctly, whereas, with severity alone, this number falls to 103 at the 0.486 threshold and to 93 at the 0.539 threshold; the second threshold also misses one of the added-band conditions. Adding the band-variance spread does not change the in-band result, while adding adjacent-band correlation raises the number of detected conditions to 113 but assigns all undisturbed scenes to the degraded class and thereby reduces the specificity to zero. The added-band column covers the undisturbed state of the seven evaluation scenes together with their thirty-five degraded conditions at five injection levels; since the striping score is not computed in that design, the corresponding rows are left empty.

Ten of the fourteen in-band conditions that escape the gate are dead lines in scenes where the removed columns are too sparse to be perceived as the column structure; the striping score reaches 0.377 in Loukia and 0.365 in the Kennedy Space Center scene but remains only in the range of 0.010–0.027 in Botswana and Pavia University. The remaining four cases occur in the Houston 2013 and Houston 2018 scenes, whose undisturbed severity is already the lowest among the ten scenes, and only at the mildest variance ratio. Signal-removing degradation, therefore, cannot be detected when it is too sparse to be seen as the spatial structure; this is a limitation of the gate rather than of the structure indicators that follow it.

At the structure classification stage, conditional on the scene having passed the degradation gate, the rule classified all eighty operating points correctly. These include the fifty-six points of the seven scenes not used in threshold calibration; no undisturbed reference scene is therefore required within the controlled standardized evaluation. What makes an absolute threshold applicable are the baseline values: in the undisturbed scenes the variance spread remained below 0.573 and, together with the adjacent-band correlation range given above, both indicators separate comfortably from their thresholds. The distances to the nearest operating point are, moreover, wide; the smallest margin is 0.048 for the striping score. This result is nevertheless conditional on the scene having been identified as degraded: it measures the structure stage only. Because of the escaping conditions noted above, the two-layer system cannot recognize every degradation. The confusion matrix is diagonal in both groups: each of the four structures was assigned correctly in all six calibration points and all fourteen evaluation points per structure, with no off-diagonal entries. Perfect classification on a finite sample, nevertheless, carries uncertainty, so the Wilson score interval is reported rather than the point estimate alone: 1.000 with a 95% interval of 0.936–1.000 on the fifty-six evaluation points, and 0.954–1.000 overall eighty points. The lower bound, therefore, admits an error rate of up to six per cent that the present sample size cannot exclude. We also note that this result is obtained on synthetically generated degradation, so it demonstrates that the indicators separate the four generators used here rather than establishing a generalizable diagnostic performance on arbitrary real degradation; the real-degradation evidence is limited to the two scenes of Section 3.2.4.

#### 3.2.4. Validation on Real Sensor Degradation

**Figure 9:**
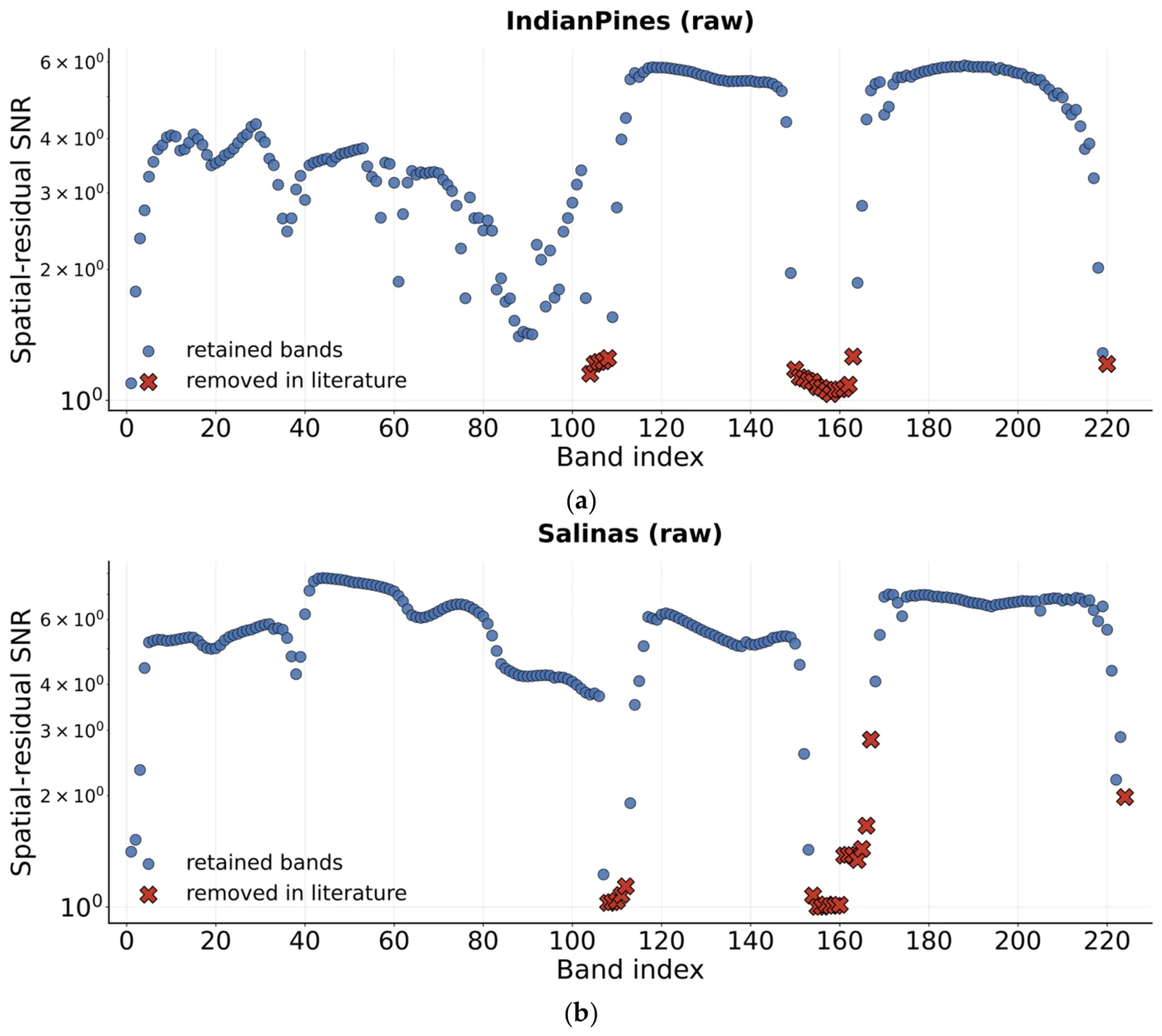
Spatial residual signal-to-noise ratio per band: (**a**) Indian Pines, and (**b**) Salinas.

In addition to the synthetic evaluation of the structure indicators, a complementary reference-free measure was tested on real sensor degradation. The spatial residual signal-to-noise ratio, estimated from horizontal neighboring-pixel differences within each band, was compared with the twenty bands conventionally removed in the literature for the Indian Pines and Salinas scenes, and the result is presented in Figure 9.

As seen in Figure 9, the measure separated the removed bands with areas under the curve of 0.997 for Indian Pines and 0.994 for Salinas. In both scenes, the twenty removed bands are indeed clustered at the lowest values of the indicator and are clearly separated from the remaining bands. Selecting the lowest twentieth percentile moreover captured all of the removed bands in both scenes. The indicator, therefore, works on real sensor degradation alone, without synthetic injection. These results lead to a two-layer diagnosis proposal in which the severity index and the structure indicators complement each other: the severity index reports how severe the degradation is and the structure indicators report of what type it is, both before classification and without labels. Together, these two layers turn the mismatch demonstrated above into action. In a scene diagnosed as striped, for example, the robustness advantage of the convolutional network disappears and the tree ensembles should be preferred.

### 3.3. Mechanism and Decision Value

**Figure 10:**
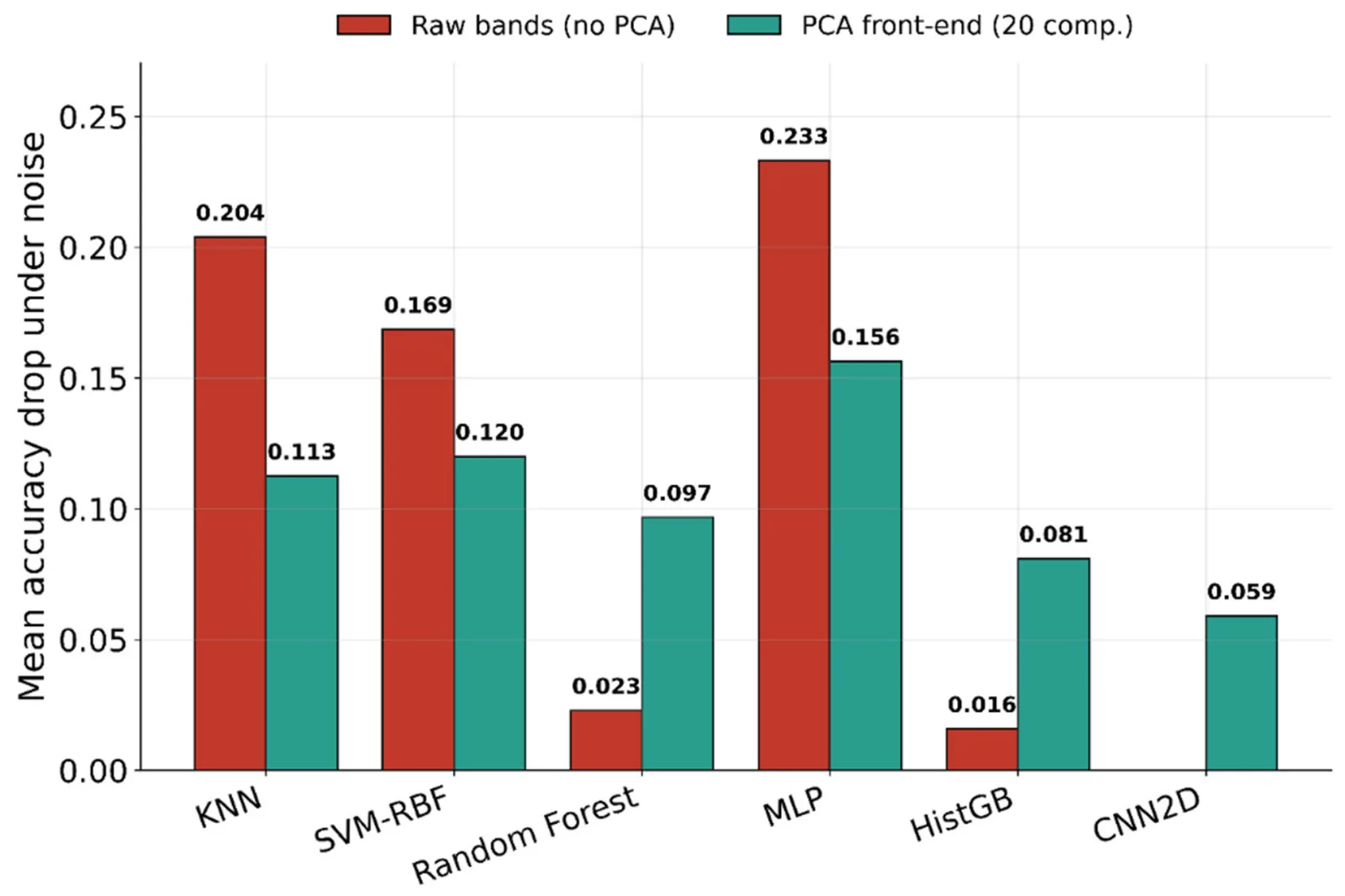
Accuracy loss under noise with and without the front end.

In order to determine whether the robustness of the convolutional network arises from its architecture or from spectral dimensionality reduction, each classical classifier was also evaluated with the same twenty-component principal component front end. The front end markedly reduced the noise sensitivity of the fragile methods: the noise loss fell from 0.204 to 0.113 for KNN, from 0.233 to 0.156 for MLP, and from 0.169 to 0.120 for SVM–RBF. A substantial part of the robustness of the convolutional network, therefore, arises not from the architecture itself but from the noise suppression provided by dimensionality reduction. Even when the front end was equalized across all methods, however, the lowest loss was still observed for the convolutional network at 0.059, with the nearest value being 0.081; the architecture, therefore, also makes an independent contribution. The comparison of the versions with and without the front end is given in Figure 10.

**Figure 11:**
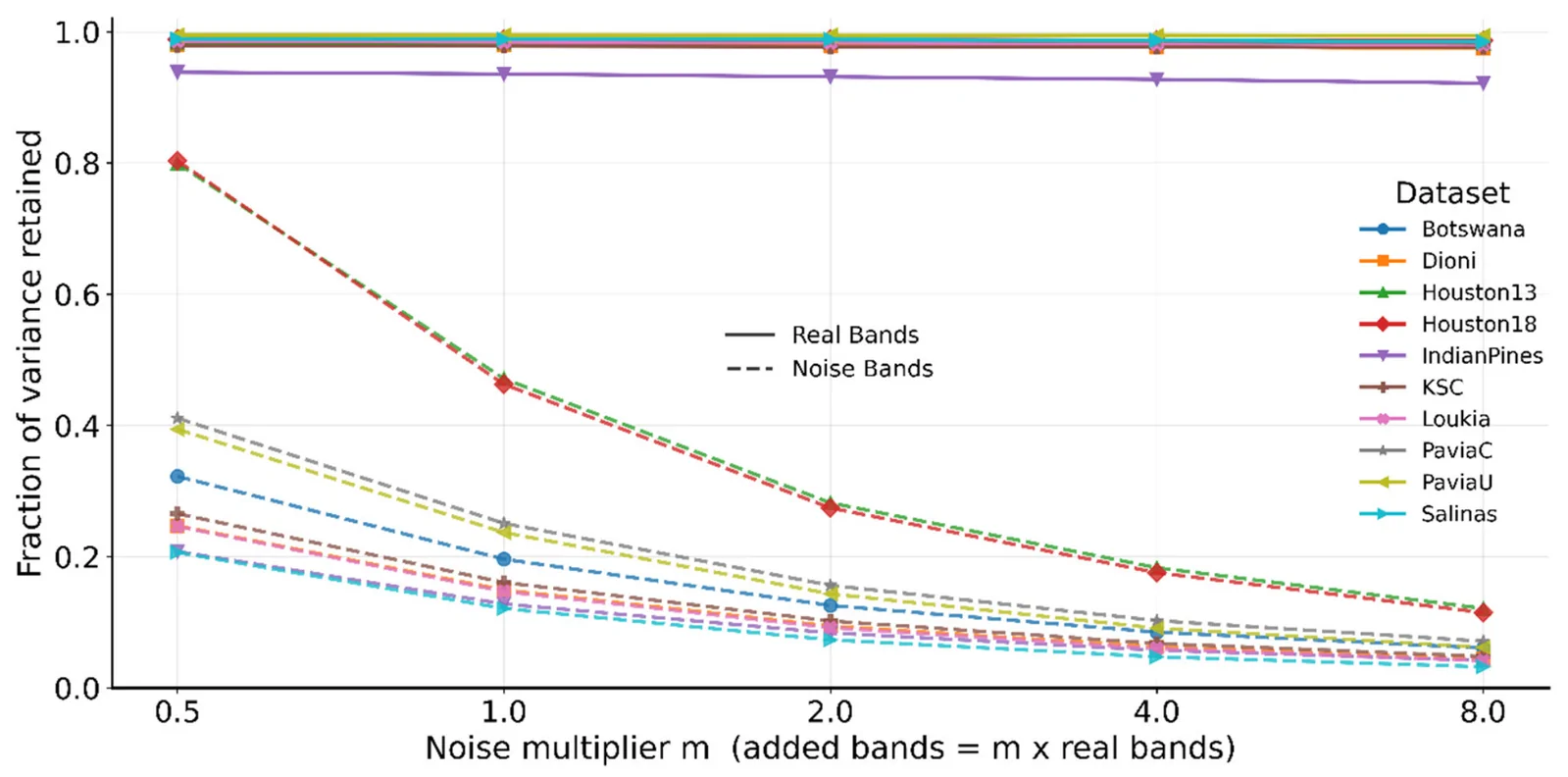
Real-band and noise-band variance retained by the front end.

Since the front end of the convolutional network is part of its architecture, no raw-band version of this method exists. For the tree ensembles, in contrast, the effect of the front end is reversed: the noise loss rose from 0.023 to 0.097 for the random forest and from 0.016 to 0.081 for HistGB. The likely reason is that tree-based methods can ignore uninformative bands through split selection, whereas the principal component transform spreads the noise across all components and thereby removes this selectivity. Indeed, the most robust methods on raw bands are the tree ensembles. The extent to which the front end of the convolutional network removes noise was measured directly. At each noise level, the proportions of variance retained by the twenty-component principal component transform, fitted only on the training pixels, were computed separately for the real bands and for the noise bands; the relationship is shown in Figure 11 with solid lines for the real bands and dashed lines for the noise bands.

The strength of the noise suppression, moreover, depends strongly on the number of bands in the scene. Computed from the curves in Figure 11, the Spearman correlation between the proportion of noise passed at the highest injection level and the original number of bands is −0.96, with a p value below 0.001. In the forty-eight-band Houston scenes, 0.115–0.121 of the noise is passed, whereas, in the Salinas and Indian Pines scenes, which have more than two hundred bands, this proportion falls to 0.032–0.042. With a fixed number of components, the filtering power of the front end inevitably weakens in scenes with few bands; scaling the number of components to the number of bands is, therefore, recommended.

**Figure 12:**
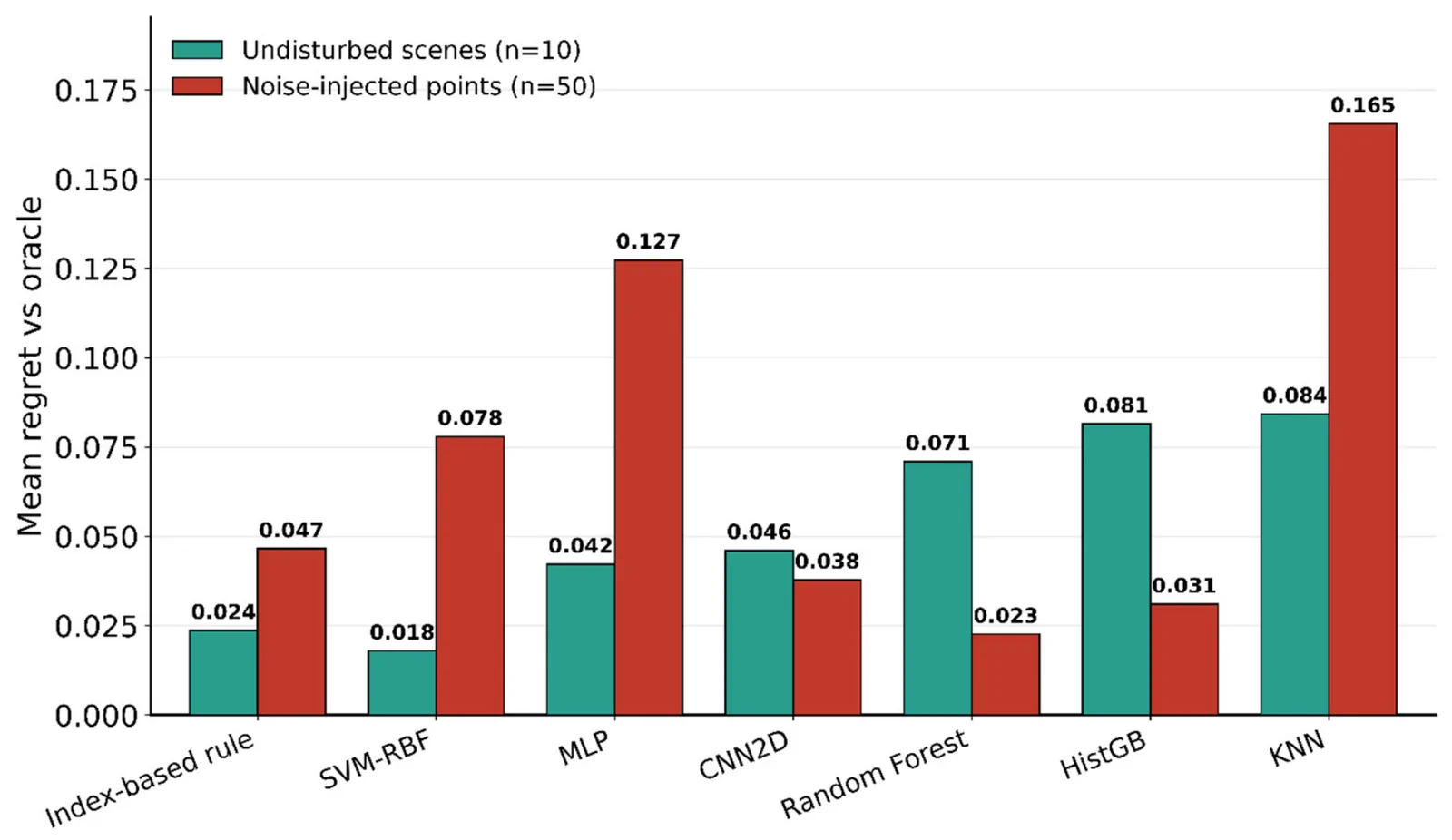
Mean regret by regime: the severity-based regression rule and fixed classifiers.

The front end behaves markedly asymmetrically: it retains between 0.922 and 0.995 of the real band variance, with a mean of 0.980, and this proportion remains almost constant irrespective of the amount of noise. The share it passes of the noise band variance, in contrast, falls from the range of 0.21–0.80 at the lowest injection level to the range of 0.032–0.121, with a mean of 0.064, at the highest. At the highest injection level, the real variance is, thus, retained between eight and thirty times more than the noise variance. This result explains the mechanism behind the behavior observed in Figure 10: the principal component front end acts as a denoiser and reduces the noise sensitivity of the fragile classifiers. For the tree ensembles, which can already ignore uninformative bands through split selection, it provides no additional gain and, instead, does harm by spreading the noise across all components. Now that the predictive power of the index for the fragile methods has been established, the question arises whether this information can be turned into a practical classifier selection rule. The severity-based regression rule of Section 2.8 was evaluated out of sample by leaving one dataset out at a time, and the comparison separated by regime is presented in Figure 12.

The rule is significantly superior to committing in advance to a fragile method: the median gain in overall accuracy is 0.101 relative to always using KNN and 0.062 relative to always using MLP, and both differences are significant under the Holm-corrected Wilcoxon test. The central finding, however, is that the best fixed classifier changes with the degradation regime. According to Figure 12, on the ten undisturbed scenes, the best fixed option is SVM–RBF with a mean regret of 0.018, while the random forest lags markedly behind at 0.071. On the fifty noise-injected operating points, in contrast, the ranking reverses; the random forest moves ahead at 0.023 and SVM–RBF rises to 0.078. Committing in advance to a single fixed classifier therefore carries a substantial cost in the wrong regime. The severity-based rule tracks this change of regime in the right direction but does not surpass the best fixed option of either regime: it yields a regret of 0.024 in the undisturbed regime and 0.047 in the noise regime. The rule is therefore presented not as a recommended optimal policy but as a direct application of the index.

**Figure 13:**
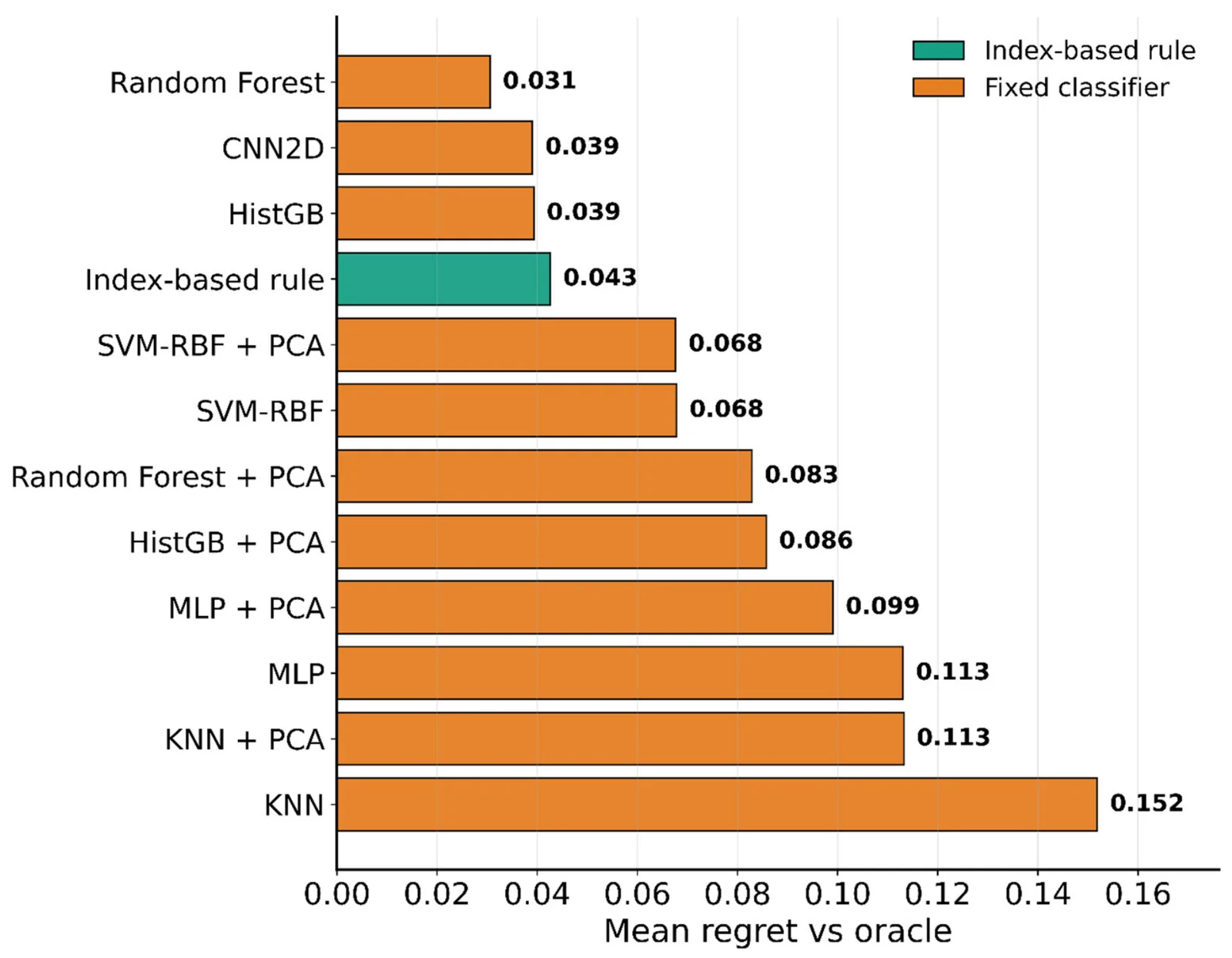
Mean regret relative to the point-wise oracle.

The performance of the rule can also be assessed with the regimes pooled. The rule is thereby compared with eleven fixed policies on a single axis; each bar shows the mean regret of the corresponding policy relative to the point-wise oracle, and lower values are better. This comparison is given in Figure 13.

Two observations stand out. First, the rule is markedly better than policies committed to fragile methods. The regret of the rule is 0.043, whereas that of always using MLP is 0.113 and that of always using KNN is 0.152, that is, between two and a half and three and a half times the regret of the rule; the index therefore eliminates the fragile options. Second, and equally importantly, the rule does not surpass the robust fixed defaults in the pooled evaluation. The regret of always using the random forest (0.031), CNN2D (0.039), or HistGB (0.039) is at the same level as that of the rule or slightly lower. Since five of the six injection levels contain synthetic noise, however, the pooled mean gives greater weight to the degraded regime. When the regimes are separated as in Figure 12, no fixed policy performs well in both conditions. The comparison therefore does not overstate the value of the rule and shows that its real contribution is not to find the best selector but to avoid the poor options.

**Figure 14:**
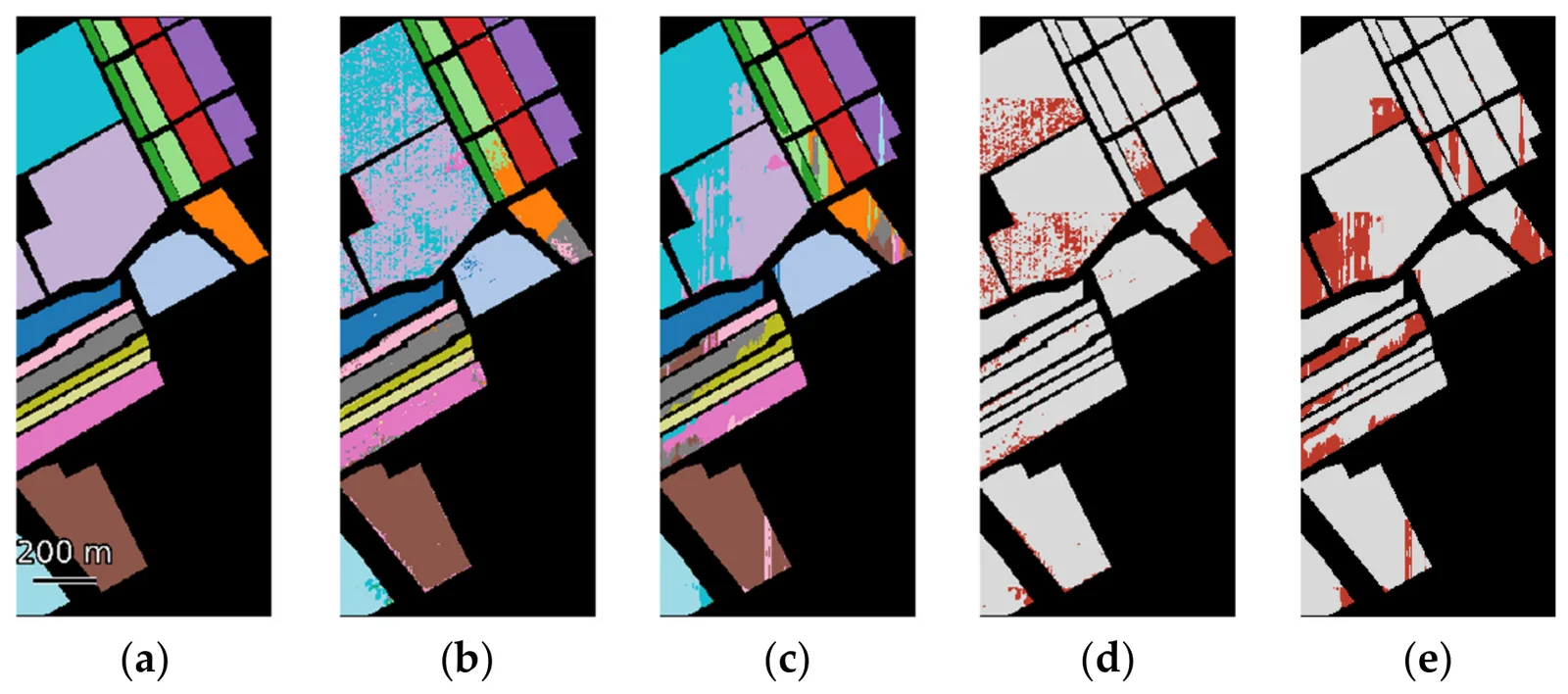
Salinas under in-band striping degradation (η=2). (**a**) reference map; (**b**) selected classifier (random forest); (**c**) avoided classifier (CNN2D); (**d**) errors of the selected classifier; (**e**) errors of the avoided classifier.

**Figure 15:**
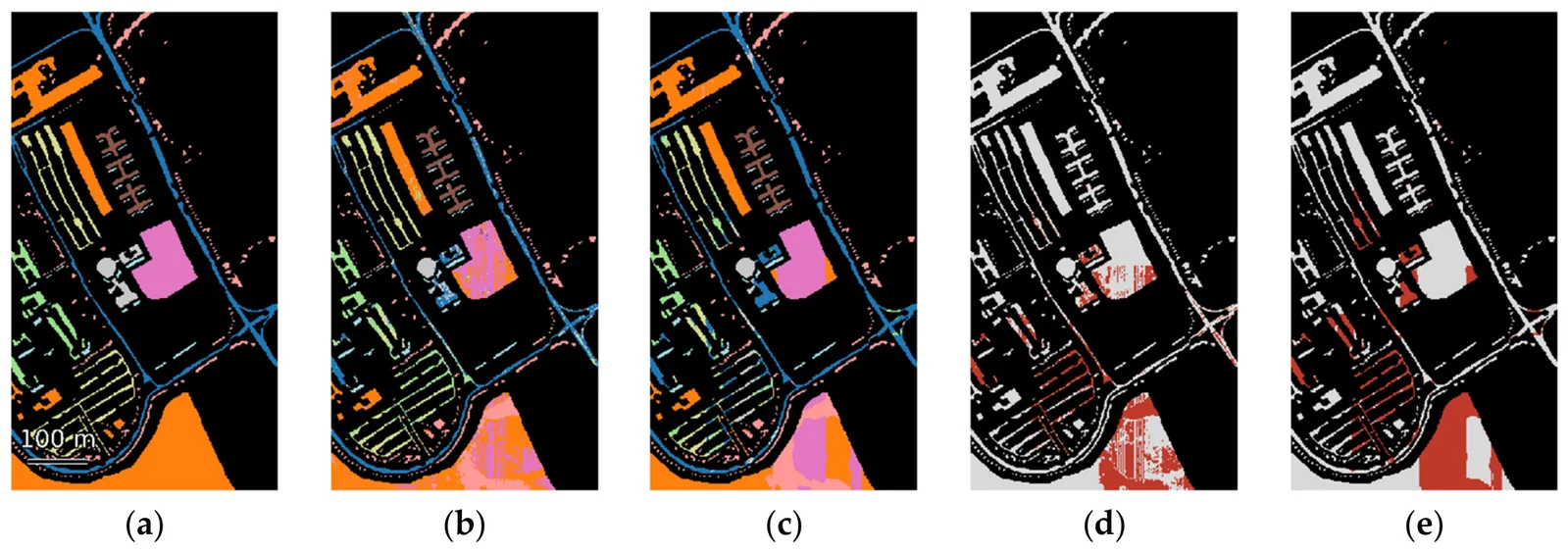
Pavia University under in-band striping degradation (η=2). (**a**) reference map; (**b**) selected classifier (random forest); (**c**) avoided classifier (CNN2D); (**d**) errors of the selected classifier; (**e**) errors of the avoided classifier.

**Figure 16:**
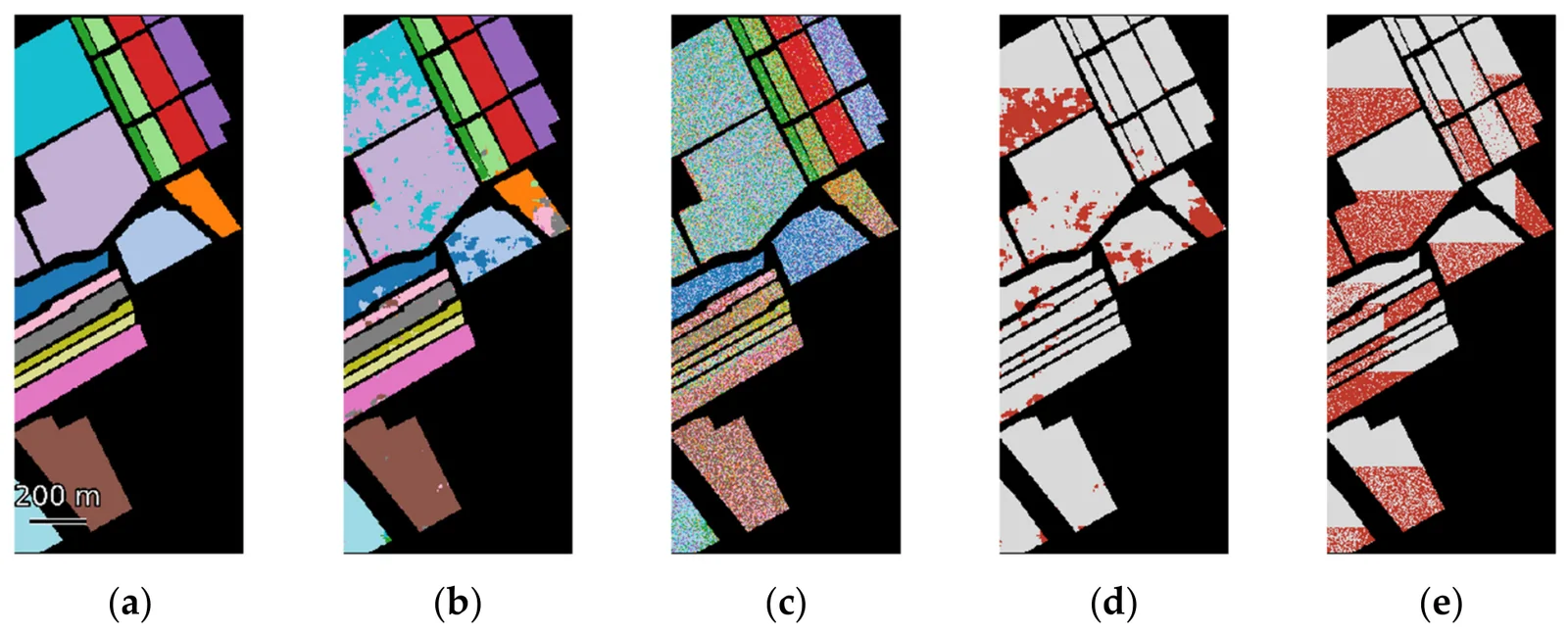
Salinas under in-band spectrally correlated degradation (η=2). (**a**) reference map; (**b**) selected classifier (CNN2D); (**c**) avoided classifier (random forest); (**d**) errors of the selected classifier; (**e**) errors of the avoided classifier.

**Figure 17:**
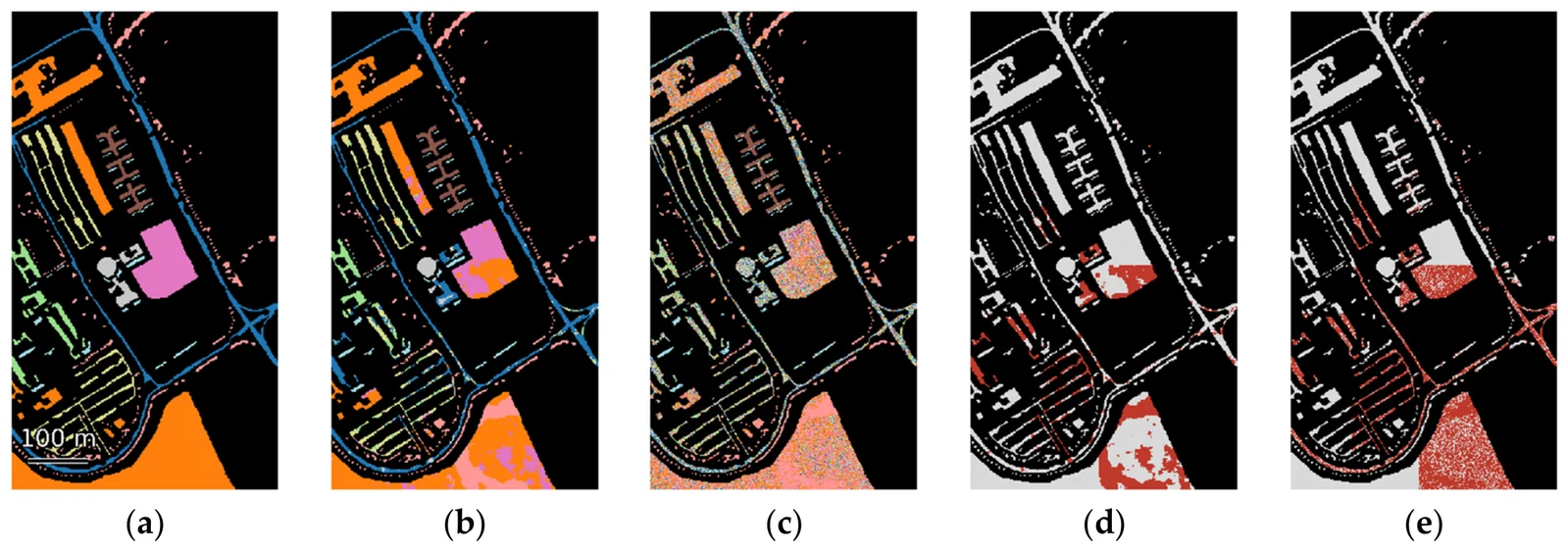
Pavia University under in-band spectrally correlated degradation (η = 2). (**a**) reference map; (**b**) selected classifier (CNN2D); (**c**) avoided classifier (random forest); (**d**) errors of the selected classifier; (**e**) errors of the avoided classifier.

What this decision value means in practice can be seen on maps. The practical consequence of the diagnosis was examined by mapping three scenes under two opposing in-band degradations following the protocol of Section 2.9. Striping selects the random forest, whose mean loss is 0.026, markedly lower than the 0.161 loss of the convolutional network. This ranking held in nine of the ten scenes, the single exception being Loukia, where the degradation improved the network very slightly. Spectrally correlated degradation, in contrast, reversed the choice: the network lost 0.062 and the random forest 0.340, and this ranking was observed in all ten scenes. In all six scene–regime combinations, the selected classifier achieved both a higher overall accuracy and a higher Kappa value. The mean advantage in overall accuracy is 0.088 under striping and 0.277 under spectrally correlated degradation. The single inconsistency is that, in Indian Pines, under striping, the average accuracy of the avoided classifier is 0.010 higher; overall accuracy and Kappa again support the selected method. It should be noted that the absolute accuracies are low in all three scenes at this severity; the comparison should therefore be read not as a claim of adequate mapping quality but as a relative comparison between the two available options. Salinas and Pavia University are shown in Figure 14, Figure 15, Figure 16 and Figure 17. The two scenes are placed side by side in each regime so that the reversal of the choice between regimes can be seen directly across scenes. The numerical results of the six combinations, including Indian Pines, are given in Table 11. The magnitude of the correction can be seen in a single example: under spectrally correlated degradation, in Salinas, the uncorrected clustering proportion reaches 0.964, whereas the proportion expected for a random placement is 0.996.

The error maps further show that the two regimes fail in different ways. Under spectrally correlated degradation, the errors of the random forest cannot be distinguished from a random placement of the same number of errors. Excess clustering is −0.03 in Salinas, 0.08 in Pavia University, and −0.06 in Indian Pines; in other words, the map degrades into pixel-level errors. Under striping, in contrast, the errors of both families are clustered in Salinas and Pavia University; this is to be expected, since the degradation itself follows the column geometry of the scene. Beyond this, the errors of the convolutional network show a clustered structure wherever clustering can be resolved, a consequence of its patch-based spatial support. In Indian Pines, in contrast, the fields are small relative to the 9 × 9 patch and the error rate is high, so the statistic remained close to zero in all conditions and the spatial structure could not be recovered. From a mapping perspective, the consequence is, therefore, that the two regimes require not only different classifiers but also different quality control methods. Pixel-level errors can be reduced by spatial filtering, whereas field-scale errors cannot be removed in this way.

**Figure 18:**
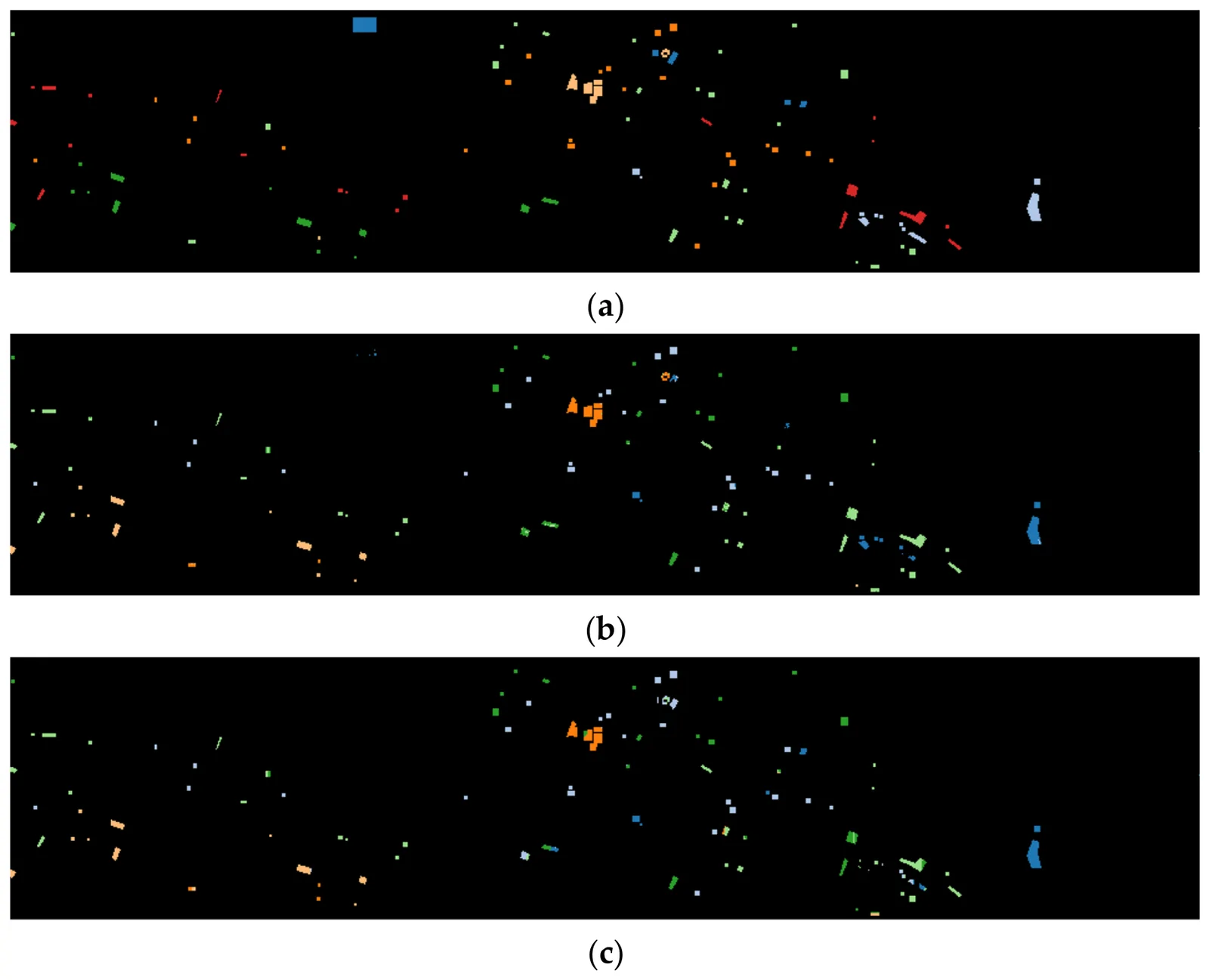

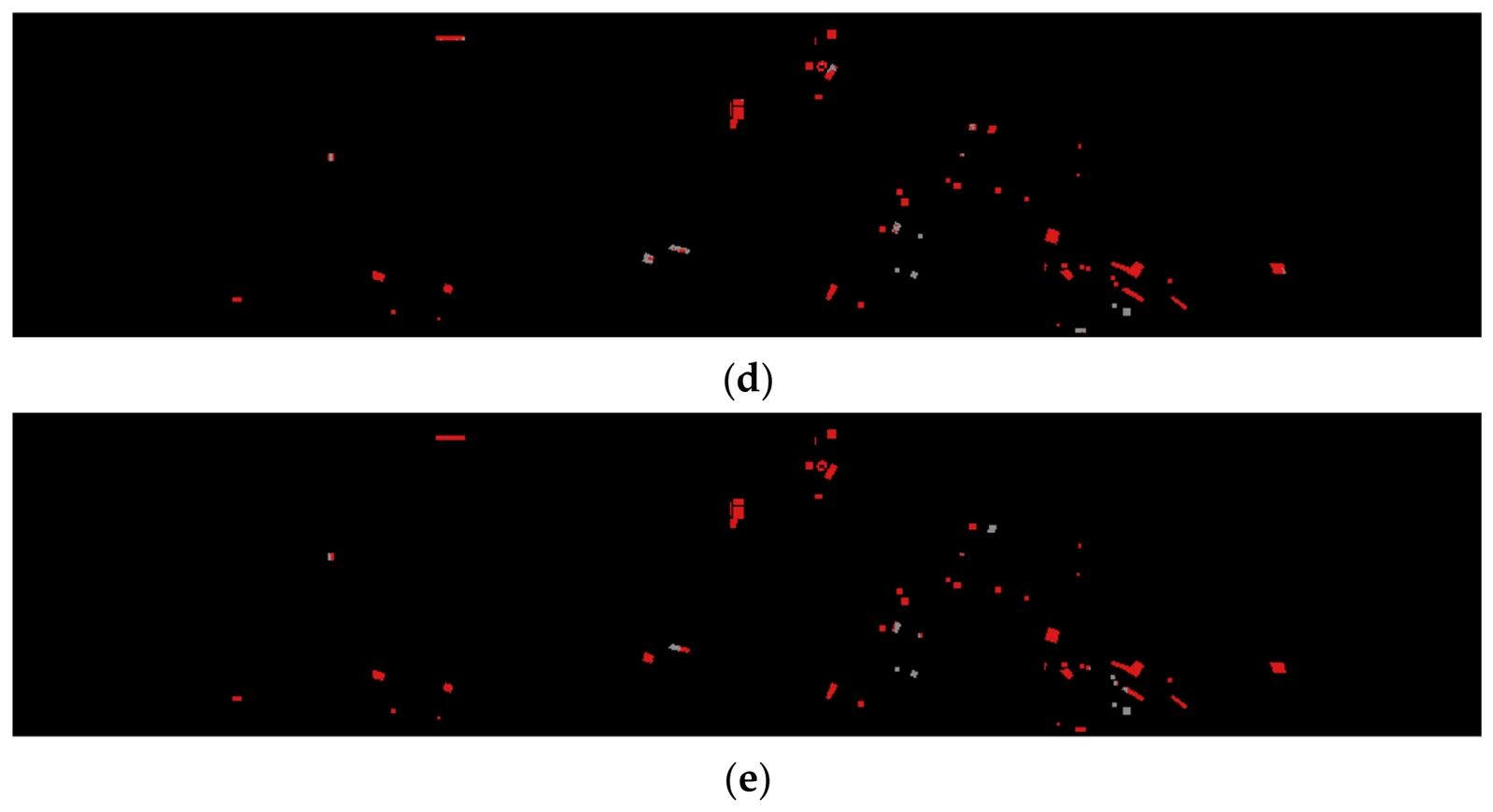
Houston 2013 under in-band striping degradation (η=2). (**a**) reference map; (**b**) selected classifier (random forest); (**c**) avoided classifier (CNN2D); (**d**) errors of the selected classifier; (**e**) errors of the avoided classifier.

**Figure 19:**
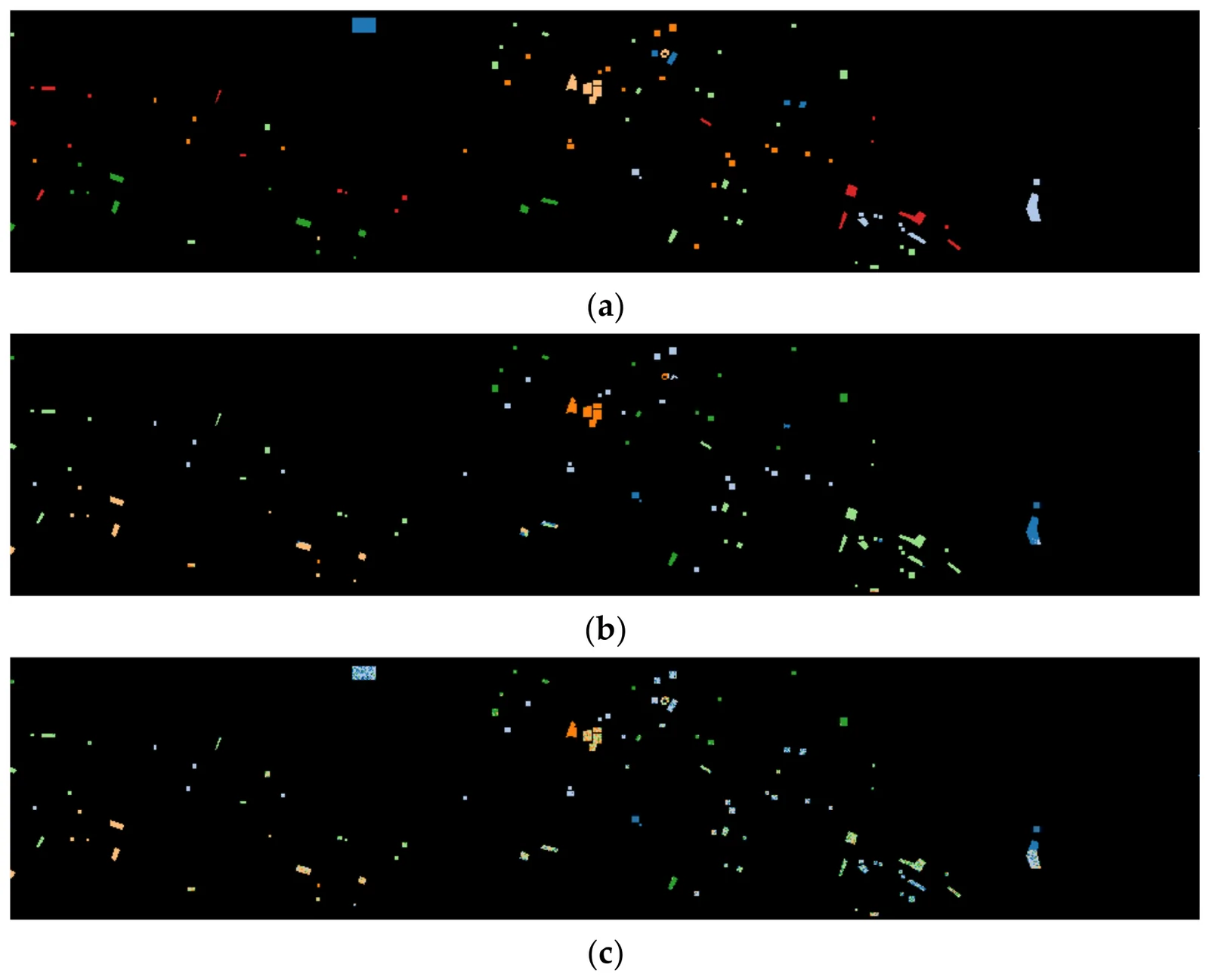

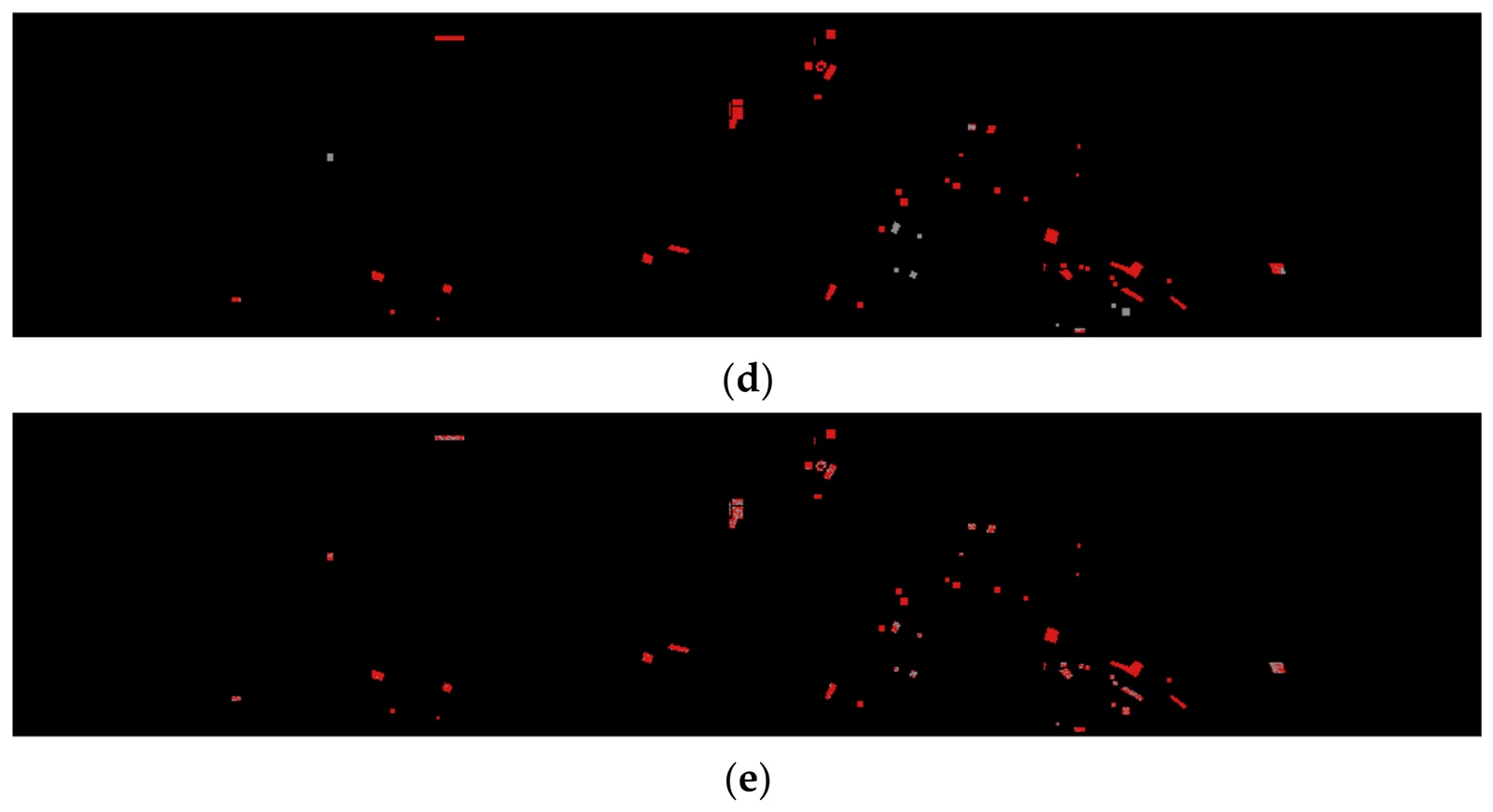
Houston 2013 under in-band spectrally correlated degradation (η=2). (**a**) reference map; (**b**) selected classifier (CNN2D); (**c**) avoided classifier (random forest); (**d**) errors of the selected classifier; (**e**) errors of the avoided classifier.

Under striping, the selected random forest reached an overall accuracy of 0.799 and a Kappa of 0.776, while the avoided convolutional network remained at 0.722 and 0.692. Since the degradation follows the column geometry of the scene, the errors of both families are clustered; excess clustering was +0.69 for the random forest and +0.92 for the convolutional network. Figure 14a, Figure 15a, Figure 16a, Figure 17a, Figure 18a and Figure 19a show the reference map, Figure 14b, Figure 15b, Figure 16b, Figure 17b, Figure 18b and Figure 19b the map of the classifier preferred in the relevant robustness regime and Figure 14c, Figure 15c, Figure 16c, Figure 17c, Figure 18c and Figure 19c the map of the avoided classifier. Figure 14d, Figure 15d, Figure 16d, Figure 17d, Figure 18d, Figure 19d, Figure 14e, Figure 15e, Figure 16e, Figure 17e, Figure 18e and Figure 19e show the errors of these two maps on the test pixels, with correctly classified pixels in grey and erroneous ones in red. Unlabelled pixels are black in all panels. Since the scenes are distributed without geolocation information, the maps are shown in sensor geometry with a metric scale bar derived from the published ground sampling distance of each sensor. No north arrow is given, in contrast, because the orientation of the scenes is not documented in their distributions.

Under the same striping degradation, the ranking is preserved in Pavia University, but the difference narrows. The random forest reached an overall accuracy of 0.572 and a Kappa of 0.483, while the convolutional network remained at 0.496 and 0.410. Since the degradation follows the column geometry of the scene, the errors of both families are clustered; excess clustering was +0.30 and +0.28, respectively.

Under spectrally correlated degradation, the choice on Salinas is reversed and the difference becomes far larger. The convolutional network reached an overall accuracy of 0.788 and a Kappa of 0.763, while the random forest fell to 0.343 and 0.291. The two failure modes also differ visually: the errors of the network remain clustered at field scale with an excess clustering of +0.84, whereas the errors of the random forest, with an excess clustering of −0.03, cannot be distinguished from a random placement and the map degrades into pixel-level noise.

Pavia University repeats the same reversal. The convolutional network reached an overall accuracy of 0.609 and a Kappa of 0.508, and the random forest 0.324 and 0.207; the excess clustering values are +0.40 and +0.08. That the reversal is observed in both scenes and in the same direction distinguishes the regime effect from a scene-specific coincidence. The demonstration so far uses the three scenes on which the thresholds were calibrated. To establish that the rule transfers, it was repeated on Houston 2013, which belongs to the evaluation group and contributed to neither the thresholds nor the family mapping. The selected and avoided classifiers were again read from the mean losses of the in-band experiment rather than chosen by hand, and the selection reversed between the two regimes exactly as it does on the calibration scenes. Under striping, the random forest was selected and reached an overall accuracy of 0.751 and a Kappa of 0.708, against 0.578 and 0.505 for the avoided convolutional network; the corresponding maps are shown in Figure 18.

Under spectrally correlated degradation, the choice reversed: the convolutional network reached 0.695 and 0.641, against 0.326 and 0.210 for the random forest, as Figure 19 shows. The selected classifier was therefore superior on the overall accuracy, average accuracy, and Kappa in both regimes, and the advantage of 0.173 and 0.369 is larger than on the calibration scenes. The excess clustering statistic is uninformative in this scene: the labelled regions of Houston 2013 are small relative to the fifty-pixel threshold, so it remains at zero in three of the four panels and reaches only 0.074 for the avoided random forest under correlated degradation. This is the same resolution limit noted for Indian Pines rather than an absence of structure in the errors.

### 3.4. Protocol and Design Stability

The accuracies reported above are lower than the values commonly given in the literature for the same scenes. It therefore has to be determined whether the difference arises from the evaluation protocol or from the implementation. The evaluation on the undisturbed data was repeated using a stratified random pixel split instead of the leakage-aware spatial split described in Section 2.6, with everything else held fixed. The result is given in Table 12. In the table, the increase column is the random value minus the spatial value, and the last column shows the mean rank under the spatial protocol followed by the rank under the random protocol. The spatial means in Table 12 were computed over the same ten splits as the last row of Table 5.

**Figure 20:**
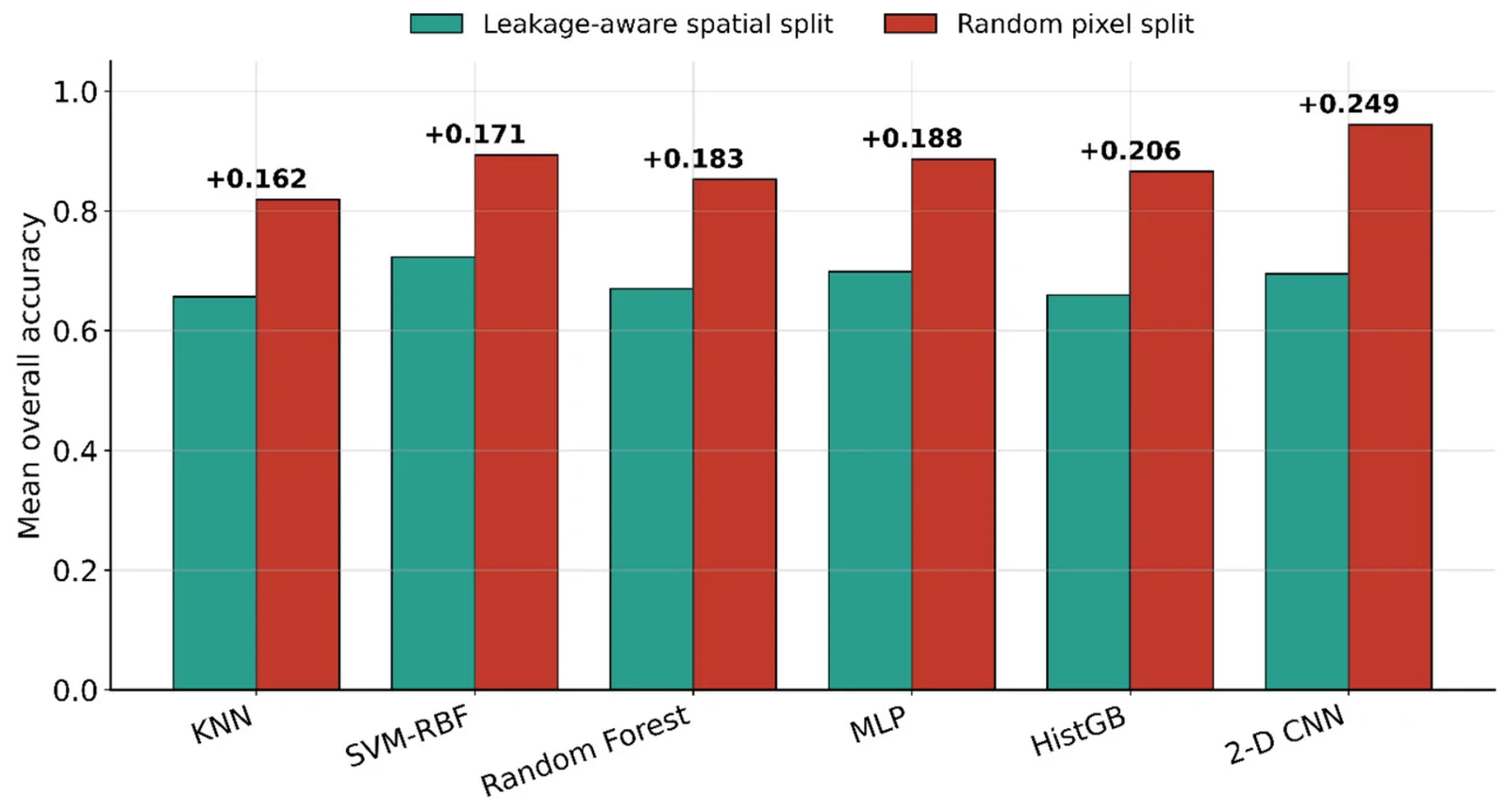
Mean overall accuracy under the two evaluation protocols.

The most important observation in Table 12 is that the increase in accuracy is not uniform across classifiers. The smallest increase, 0.162, occurs for the nearest neighbor classifier and the largest, 0.249, for the convolutional network; the protocol therefore favors the patch-based model most. The mechanism is clear: in a random split, the patch surrounding a test pixel often contains training pixels. Part of the spatial context on which the network relies has thereby been seen during training together with its label. This leakage mechanism and its consequences for benchmark splits have been documented by Nalepa, Myller, and Kawulok [55]. The effect is also clearly visible in the ranking: the mean rank of the convolutional network improves from 3.0 under the spatial protocol to 1.3 under the random protocol, while SVM–RBF falls from 1.5 to 2.0. The best classifier changes in six of the ten scenes: under the random split, the convolutional network is best in nine scenes, whereas, under the spatial split, SVM–RBF is best in five scenes and the convolutional network in four. The Spearman correlation between the two sets of mean ranks is 0.73 and is not significant at conventional levels, with a *p* value of 0.10. It therefore cannot be said that the two protocols merely produce the same ranking at a different scale. The random protocol does not only shift the reported accuracy level but also changes which method appears best, in favor of the architectures that benefit most from the spatial dependence. This is a further reason for reading comparative results based on random pixel splits with care, and it is the reason the leakage-aware protocol was used throughout this study. The same results are also presented per classifier in Figure 20.

Under the random split, the mean overall accuracy rises from 0.656 to 0.819 for KNN, from 0.723 to 0.893 for SVM–RBF, from 0.670 to 0.852 for the random forest, from 0.699 to 0.886 for MLP, from 0.659 to 0.866 for HistGB, and from 0.695 to 0.944 for the convolutional network. This increase was observed for all six classifiers in all ten scenes, and the Wilcoxon signed-rank test over the ten scenes gave *p* = 0.002 for every classifier, the smallest value attainable at this sample size. The magnitude of the increase nevertheless depends on the scene: averaged over the classifiers, it is 0.041 in Pavia Centre, where the labelled classes form large homogeneous regions, and 0.323 in Indian Pines, where the fields are small and the labelled pixels densely interleaved. The present pipeline, thus, reproduces, under a random split, the accuracy range usually reported in the literature. Indeed, the convolutional network reached an accuracy of 1.000 in the Kennedy Space Center scene, 0.999 in Botswana and Houston 2013, 0.993 in Pavia Centre, and 0.960 in Salinas. The low values in Table 5 are, therefore, a property of the protocol rather than of the implementation. Because p=0.002 is the smallest value attainable with ten paired scenes, it is reported without Holm correction as an exact bound rather than as a comparison between classifiers; the six tests address six separate questions and are not competing hypotheses about a single effect.

**Figure 21:**
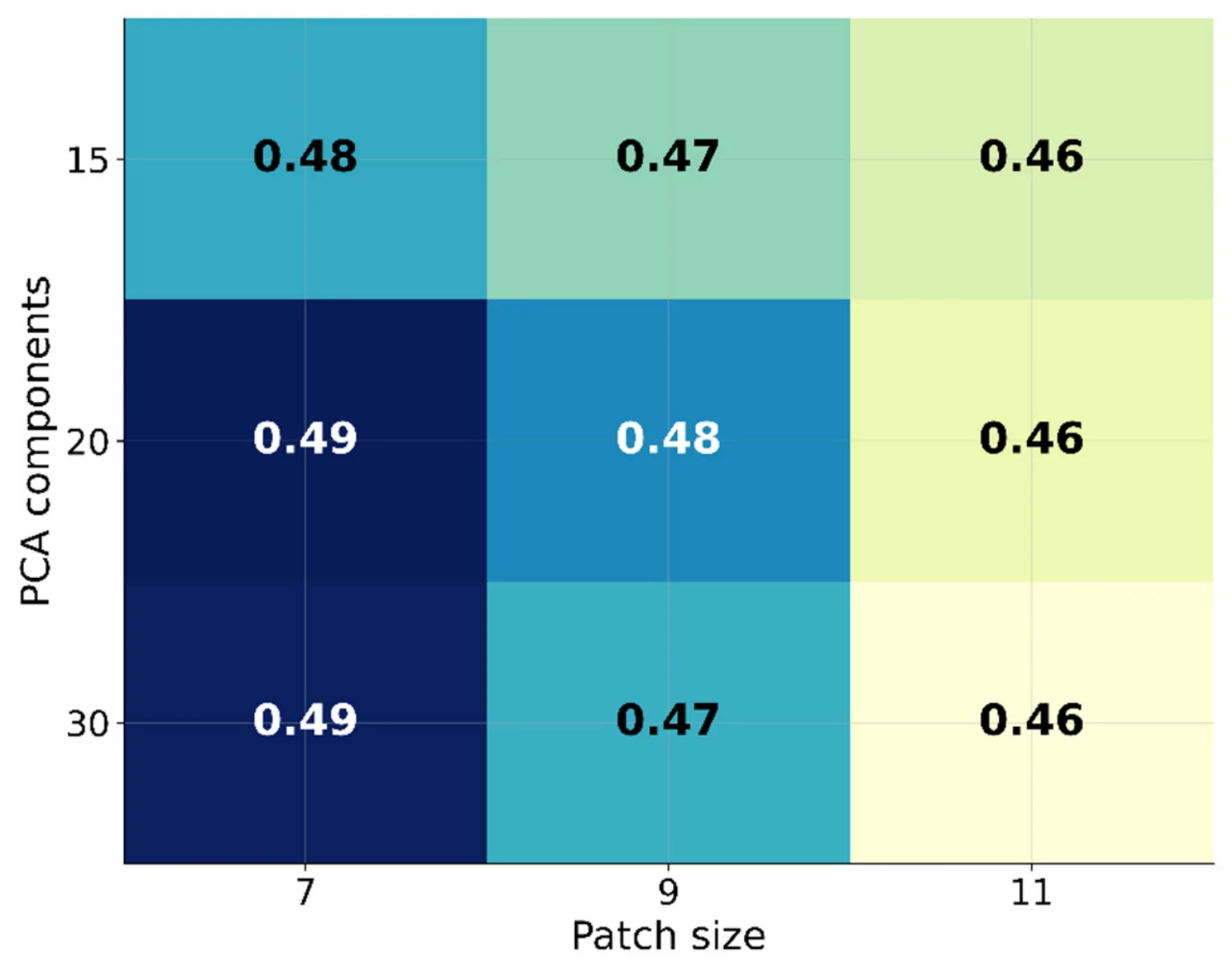
CNN2D sensitivity to the number of principal components and the patch size.

Since the two-regime finding rests on this design choice, one further element has to be checked: whether the behavior of the convolutional network is stable across its own hyperparameters. To this end, the sensitivity analysis carried out for the number of principal components (15, 20, and 30) and the patch size (7, 9, and 11) is given in Figure 21. In this analysis, the width of the spatial buffer was fixed to the largest patch size in all grid cells. Otherwise, the buffer depends by default on the patch size, so that the training–test split changes as the patch changes and the measured effect becomes a mixture of patch size and split difficulty.

The grid in Figure 21 behaves regularly once the buffer is fixed: accuracy falls very slightly as the patch size increases, with marginal means of 0.487 for seven, 0.474 for nine, and 0.460 for eleven; the effect of the number of principal components is smaller still, with marginal means remaining in the range of 0.469–0.477. Each cell was moreover averaged over three splits, and the within-cell standard deviations lie between 0.03 and 0.05. The difference between the highest and the lowest value in the grid is 0.036, of the same order of magnitude as the mean within-cell standard deviation of 0.037 across seeds. The observed differences therefore cannot be distinguished from repetition noise, and the performance of the convolutional network is stable across the hyperparameter range examined. It should be emphasized that this stability is visible only when the buffer is fixed: when the buffer is varied together with the patch size, the total variation in the same grid rises to 0.191, although that difference arises from the split difficulty rather than from patch size. It should also be borne in mind that the results were obtained on Indian Pines, a comparatively difficult scene, and with an architecture deliberately kept simple in order to preserve the controlled comparison.

## 4. Discussion

Three findings of this study bear directly on how classifier families should be selected and compared for hyperspectral image classification. The classifiers evaluated here are general machine-learning families applied to hyperspectral imagery rather than algorithms specific to it; what is hyperspectral is the input, namely, its spectral band structure and the degradation that structure carries. The first is that, in the setting examined here, the effective variable is not nominal dimensionality: adding redundant bands leaves the accuracy essentially unchanged, whereas mixing the same amount of variance into the existing bands is, on average, more harmful across classifiers and degradation structures. What matters is, therefore, where the uninformative variance is placed rather than how much of it there is. The second finding is that no classifier family is robust across all degradation structures. Tree ensembles withstand spatially local degradation but not degradation spread across the spectrum, while the convolutional network behaves in the opposite way. The map-level comparison shows that this distinction is not merely numerical: the regime-based classifier selection produced the better map in all six scene-regime combinations examined. The third finding is that the evaluation protocol determines both the reported accuracy level and the ranking of the methods. This means that a robustness claim can be interpreted only together with the protocol under which it was obtained. Taken together, the findings support treating degradation diagnosis not as an analysis performed afterwards but as a step that precedes classifier selection.

### 4.1. Interpretation of the Mechanism

The controlled experiment does not contradict the Hughes peaking phenomenon [3] but, instead, identifies the effective variable. Since intrinsic dimensionality lies only between 3.76 and 8.55 while the nominal number of bands ranges from 48 to 204, adding dimensions within the spanned subspace neither increases the distance concentration nor degrades the accuracy; what produces these two effects is solely variance independent of the class structure. The robustness difference between classifiers, in contrast, arises from how each method handles uninformative directions. Nearest neighbors and the RBF kernel, for example, depend directly on Euclidean distances, which concentrate under noise, while MLP tends to learn from uninformative inputs. Tree ensembles, by contrast, can ignore entire noise bands through implicit feature selection but cannot exclude noise mixed into informative bands. Similarly, while the convolutional network removes low-variance spread noise through its principal component front end, it remains exposed to spatially structured noise that the convolution layers read as signal. One practical implication is that the value of dimensionality reduction in hyperspectral data lies not only in compression but also in denoising; this observation is consistent with the restoration literature reviewed by Rasti et al. [56]. Band selection, on the other hand, is mostly framed not as a response to noise but as the problem of removing spectral redundancy while retaining informative bands [7]. An explicit noise-awareness criterion therefore appears to be a gap in the literature worth addressing.

The distinction stated in the Introduction can be assessed in the light of the findings. Studies that measure robustness afterwards [25,27] report how models behave when the type of degradation is known. The results obtained here show that this information can also be produced before classification: the structure indicators identified the type of degradation correctly at every operating point that passed the degradation gate. The separation is nevertheless not complete; since the gate misses sparse signal-removing degradation, the diagnosis cannot be established under all conditions. The difference from reference-free quality estimation approaches [28] lies in the quantity measured: what is targeted here is not the perceptual quality of the image but which classifier family is at risk. The observation that the size of the spatial support determines robustness [26] points in the same direction as the fragility of the convolutional network under striping. The two approaches are also validated against different targets. A reference-free quality metric is validated against human judgement or against a distortion applied to a clean reference, so its success criterion is agreement with the perceived quality. The indicators proposed here are validated against the identity of the degradation generator and, in the map-level demonstration, against the classification outcome; their success criterion is whether the classifier chosen on their basis produces the better map. A high-quality scene by the perceptual criterion may still contain the spatially structured degradation that makes a patch-based network the wrong choice, and this is the case the present indicators are designed to detect.

The practical implication of the findings is the following: the actionable contribution of the study is not the rule itself but the demonstration that the ranking of classifiers depends on the degradation regime and that a label-free indicator can flag fragility before classification. The practical recommendation is accordingly that a kernel-based classifier be preferred in scenes with limited degradation, and a robust default such as a tree ensemble or a spectral–spatial convolutional network where noise is pronounced. Distance- and kernel-based classifiers should be avoided when the severity index is high. It should also be borne in mind that the noise regime was created by synthetic injection and that the highest levels correspond to extreme conditions not expected in practice. The leakage-aware split is central to this honesty: the absolute accuracies reported here are lower than the results from random pixel splits, which are inflated by spatial dependence [36].

### 4.2. Limitations

The scope of the experimental design is limited in several respects. The added-band sweep rests on idealized independent Gaussian noise, the structured noise variant is confined to three scenes, and the highest injection levels correspond to extreme conditions not expected in practice. The trend across datasets is, moreover, weak, a single modest convolutional architecture was used without evaluating transformer and state-space architectures, and the intrinsic dimensionality estimate depends on the estimator chosen.

Two qualifications are required as regards the independence of the indicators. The striping indicator and its threshold were revised after the first version failed on three scenes with a strong natural column structure. For this indicator, the evaluation on the scenes excluded from numerical threshold calibration is, therefore, not as independent as that of the other two indicators. The diagnostic rule directly targets four basic degradation structures, whereas the robustness experiments include six in-band structures and the real degradation validation covers two scenes.

A qualification also applies as regards reference-freeness. The band-variance spread is computed on a cube standardized before the degradation is added. Its transfer to the raw radiance that a new scene would provide has therefore not been tested here. While the severity and striping gate captures degradation that adds variance or creates an unshared column structure, it misses the signal removal too sparse to create such a structure, as seen in 14 of the 120 in-band conditions.

The scope within which the findings may be interpreted is likewise limited. The results obtained show that embedding a low-dimensional subspace in a higher nominal dimension is harmless; this should not, however, be interpreted as the broader claim that high dimensionality is harmless in general.

Finally, the map-level demonstration is limited to three scenes, a single severity levelm and a single split, and uses a simplified convolutional network. The three scenes used are the same as those on which the thresholds were calibrated; the demonstration, therefore, shows what the regime distinction means for map quality rather than constituting an independent test. Because the degradation was, moreover, applied to the entire scene before splitting, a single realization of the stripes is shared between the training and test regions, which means that the model may partly learn the artefact rather than only being harmed by it. Independent realizations for training and testing could separate these two effects. That separation, together with the extension of the controlled protocol to additional sensors and a wider set of real degradations, is left to future work. The demonstration on Houston 2013 removes the dependence on the calibration scenes but not the dependence on synthetic degradation: the striping and correlated noise applied there are generated by the same injection models used throughout. A demonstration on degradation that is, itself, of sensor origin would be a stronger test of the selection rule, and the evidence currently available for real degradation is confined to the band-level measure of Section 3.2.4 on two scenes.

## 5. Conclusions

In this study, starting from the problem that classifier selection depends on the degradation in the scene while that degradation cannot be measured without labels at the moment of selection, a two-layer degradation diagnosis computed before classification and without labels has been proposed.

A controlled experiment on ten hyperspectral datasets and six classifiers first separated dimensionality from noise: within the low intrinsic dimensional scenes and the redundant band structure examined here, adding informative but redundant bands did not change accuracy appreciably. Beyond this, comparing the addition of the same variance as separate bands with mixing it into the existing bands at a matched severity showed in-band degradation to be more harmful, on average, across classifiers and degradation structures, with the mean difference in the overall accuracy per classifier reaching 0.110. The effective quantity is, therefore, not only the amount of uninformative variance but where that variance is placed and how it is structured.

The classifier fragility accordingly follows two regimes. Tree ensembles are robust to spatially local degradations such as striping and deadlines, but as fragile as the distance- and kernel-based methods once the degradation is spread across the spectrum. The convolutional network, in contrast, is robust to spread noise as a result of its principal component front end and fragile under striping. Since no family is robust across all noise structures, the diagnosis has to report not only the magnitude of the degradation but also its type.

The severity index based on distance concentration flags the noise sensitivity of the fragile families, and its generalization is assessed with the stricter leave-one-dataset-out protocol. The three scene-derived structure indicators, calibrated on three scenes, classified correctly all fifty-six structure-labelled operating points across the seven evaluation scenes within the controlled standardized setting and without requiring an undisturbed reference scene. A complementary reference-free band-level signal-to-noise measure, moreover, reached areas under the curve of 0.994 and 0.997 in identifying the conventionally removed bands in two real scenes. The selection rule accompanying the diagnosis reliably avoids the fragile methods but does not surpass the best fixed option of each regime; indeed, the best fixed classifier is itself regime-dependent. The actionable contribution is, therefore, not a single selection rule but the demonstration that a label-free diagnosis can report both the level and the structure of the degradation before classification, and that classifier selection should follow the noise regime. Throughout, the label-free property concerns the diagnosis and the selection rule rather than the classification pipeline as a whole, which remains supervised.

The contribution of the evaluation protocol itself was isolated by repeating the undisturbed evaluation under a random pixel split with everything else held fixed. Accuracy was inflated by between 0.162 and 0.249, most of all for the patch-based network, and the best classifier changed in six of the ten scenes. The protocol was thereby shown to determine not only the reported accuracy level but also the ranking of the methods.

## Figures and Tables

**Table 1:** Properties of the ten datasets.

Dataset	Pixels	Classes	Bands
Indian Pines	10,249	16	200
PaviaU	42,776	9	103
PaviaC	39,355	7	102
Salinas	54,129	16	204
KSC	5211	13	176
Botswana	3248	14	145
Dioni	20,024	12	176
Loukia	13,503	14	176
Houston 2013	2530	7	48
Houston 2018	53,200	7	48

**Table 2:** Label-free diagnostic measures of the ten datasets.

Dataset	Intrinsic Dim.	$L_{red}$	$L_{conc}$	RC	Hubness Skewness
Indian Pines	8.55	0.957	0.486	3.70	1.00
PaviaU	5.44	0.947	0.260	5.49	0.35
PaviaC	5.80	0.943	0.444	3.59	0.43
Salinas	6.41	0.969	0.460	2.52	0.72
KSC	6.52	0.963	0.440	3.43	0.62
Botswana	6.97	0.952	0.316	6.01	0.41
Dioni	8.18	0.954	0.351	5.00	0.80
Loukia	8.28	0.953	0.313	5.68	1.24
Houston 2013	3.76	0.922	0.213	11.88	0.04
Houston 2018	3.83	0.920	0.234	4.07	0.02

**Table 3:** Accuracy loss from the lowest to the highest injection level.

Classifier	Redundancy Loss	Noise Loss	Noise Contribution	Slope	*R* ^2^
KNN	−0.018	+0.204	+0.221	−0.316	0.226
SVM–RBF	−0.003	+0.169	+0.171	−0.263	0.169
Random forest	−0.020	+0.023	+0.043	−0.053	0.007
MLP	−0.004	+0.233	+0.237	−0.371	0.294
HistGB	−0.041	+0.016	+0.057	−0.018	0.001
CNN2D	−0.007	+0.059	+0.066	−0.062	0.009

**Table 4:** Difference between in-band and added-band accuracy loss at matched severity.

Classifier	Homosc.	Band-Dep.	Correlated	Striping	Dead Lines	Mixed
KNN	+0.032	−0.024	+0.202	+0.117	+0.130	+0.080
SVM–RBF	+0.068	−0.000	+0.245	+0.097	+0.121	+0.127
Random forest	+0.138	+0.019	+0.261	+0.012	+0.004	+0.165
MLP	+0.054	−0.017	+0.228	+0.081	+0.126	+0.117
HistGB	+0.143	+0.019	+0.256	+0.019	+0.003	+0.171
CNN2D	−0.039	−0.031	−0.005	+0.073	+0.085	−0.023

**Table 5:** Overall accuracy of the six classifiers on undisturbed data.

Dataset	KNN	SVM–RBF	RF	MLP	HistGB	CNN2D
Indian Pines	0.413 ± 0.032	0.524 ± 0.032	0.441 ± 0.023	0.518 ± 0.058	0.470 ± 0.025	0.442 ± 0.081
Pavia University	0.579 ± 0.043	0.687 ± 0.082	0.595 ± 0.051	0.654 ± 0.052	0.588 ± 0.041	0.729 ± 0.181
Pavia Centre	0.879 ± 0.016	0.909 ± 0.018	0.872 ± 0.016	0.893 ± 0.024	0.873 ± 0.024	0.926 ± 0.038
Salinas	0.753 ± 0.022	0.762 ± 0.027	0.746 ± 0.032	0.757 ± 0.024	0.753 ± 0.029	0.797 ± 0.035
KSC	0.672 ± 0.035	0.726 ± 0.021	0.649 ± 0.051	0.662 ± 0.054	0.655 ± 0.059	0.796 ± 0.029
Botswana	0.778 ± 0.015	0.806 ± 0.017	0.751 ± 0.017	0.782 ± 0.024	0.724 ± 0.029	0.714 ± 0.083
Dioni	0.582 ± 0.039	0.685 ± 0.040	0.596 ± 0.055	0.658 ± 0.036	0.588 ± 0.057	0.660 ± 0.059
Loukia	0.567 ± 0.028	0.601 ± 0.033	0.602 ± 0.030	0.591 ± 0.031	0.588 ± 0.036	0.445 ± 0.038
Houston 2013	0.745 ± 0.056	0.814 ± 0.046	0.795 ± 0.040	0.763 ± 0.052	0.698 ± 0.042	0.745 ± 0.062
Houston 2018	0.597 ± 0.038	0.714 ± 0.058	0.650 ± 0.049	0.708 ± 0.049	0.656 ± 0.049	0.692 ± 0.061
Mean	0.656	0.723	0.670	0.699	0.659	0.695

**Table 6:** Severity-accuracy relationship over pooled operating points: correlation, slope, and out-of- sample R^2^.

Classifier	Spearman ρ	*p*	Slope	*R*^2^ (LODO)
KNN	−0.438	<0.001	−0.316	+0.040
SVM–RBF	−0.415	0.001	−0.263	−0.039
MLP	−0.550	<0.001	−0.371	+0.125
Random forest	−0.172	0.189	−0.053	−0.244
HistGB	−0.082	0.534	−0.018	−0.249
CNN2D	−0.078	0.552	−0.062	−0.225

**Table 7:** Severity-accuracy slope from the mixed effects model and the dataset-level cluster bootstrap.

Classifier	Mixed Model Slope	SE	*p*	Bootstrap Slope	95% Confidence Interval	*p*
KNN	−0.350	0.044	<0.001	−0.316	(−0.418, −0.226)	<0.001
SVM–RBF	−0.286	0.032	<0.001	−0.263	(−0.381, −0.168)	<0.001
MLP	−0.396	0.027	<0.001	−0.371	(−0.471, −0.288)	<0.001
Random forest	−0.038	0.009	<0.001	−0.053	(−0.151, +0.027)	0.254
HistGB	−0.027	0.010	0.006	−0.018	(−0.104, +0.052)	0.665
CNN2D	−0.103	0.042	0.014	−0.062	(−0.162, +0.030)	0.193

**Table 8:** Leave-one-dataset-out prediction error of the severity index.

Classifier	MAE	RMSE	*R*^2^ (LODO)	90% Interval
KNN	0.108	0.127	+0.040	(−0.19, +0.22)
SVM–RBF	0.106	0.127	−0.039	(−0.23, +0.25)
MLP	0.103	0.125	+0.125	(−0.18, +0.25)
Random forest	0.114	0.137	−0.244	(−0.26, +0.23)
HistGB	0.100	0.125	−0.249	(−0.23, +0.24)
CNN2D	0.119	0.144	−0.225	(−0.21, +0.26)

**Table 9:** Accuracy loss under in-band degradation (η=2).

Classifier	Homosc.	Band-Dep.	Correlated	Striping	Deadlines	Mixed
KNN	0.197	0.105	0.368	0.291	0.223	0.252
SVM–RBF	0.202	0.106	0.395	0.232	0.206	0.274
Random forest	0.196	0.037	0.340	0.026	0.008	0.233
MLP	0.201	0.104	0.382	0.220	0.204	0.276
HistGB	0.202	0.035	0.336	0.034	0.007	0.241
CNN2D	0.032	0.031	0.062	0.161	0.152	0.055

**Table 10:** Performance of the degradation gate under different configurations.

Gate Configuration	Added-Band Conditions	In-Band Conditions	Undisturbed Scenes
Severity (0.486) + striping (0.248)	42/42	106/120	10/10
Severity only (0.486)	42/42	103/120	10/10
Severity only (0.539)	41/42	93/120	10/10
Band-variance spread added	-	106/120	10/10
Adjacent-band correlation added	-	113/120	0/10

**Table 11:** Map-level comparison of the two classifiers (η=2).

Scene	Degradation	Selected	OA/kappa	Avoided	OA/kappa	Excess Clustering
Salinas	Striping	RF	0.799/0.776	CNN2D	0.722/0.692	+0.69/+0.92
PaviaU	Striping	RF	0.572/0.483	CNN2D	0.496/0.410	+0.30/+0.28
Indian Pines	Striping	RF	0.395/0.325	CNN2D	0.284/0.205	−0.05/+0.00
Salinas	Spectral corr.	CNN2D	0.788/0.763	RF	0.343/0.291	+0.84/−0.03
PaviaU	Spectral corr.	CNN2D	0.609/0.508	RF	0.324/0.207	+0.40/+0.08
Indian Pines	Spectral corr.	CNN2D	0.382/0.306	RF	0.281/0.197	+0.01/−0.06

**Table 12:** Effect of the evaluation protocol on undisturbed results.

Classifier	OA, Spatial	OA, Random	Increase	Range	*p*	Mean Rank
KNN	0.656	0.819	0.162 ± 0.081	0.027–0.254	0.002	4.7 to 5.9
SVM–RBF	0.723	0.893	0.171 ± 0.071	0.033–0.283	0.002	1.5 to 2.0
Random forest	0.670	0.852	0.183 ± 0.084	0.046–0.318	0.002	4.3 to 4.9
MLP	0.699	0.886	0.188 ± 0.082	0.026–0.291	0.002	2.8 to 3.1
HistGB	0.659	0.866	0.206 ± 0.094	0.046–0.320	0.002	4.7 to 3.8
CNN2D	0.695	0.944	0.249 ± 0.113	0.066–0.494	0.002	3.0 to 1.3

## Data Availability

The data presented in this study are available from the corresponding author upon reasonable request.

## Abbreviations

The following abbreviations are used in this manuscript:

CNN2D	Two-dimensional Convolutional Neural Network
HistGB	Histogram-based Gradient Boosting
KNN	k-Nearest Neighbor
KSC	Kennedy Space Center
LODO	Leave-One-Dataset-Out
MLP	Multi-Layer Perceptron
OA	Overall Accuracy
PaviaC	Pavia Centre
PaviaU	Pavia University
RC	Relative Contrast
SE	Standard Error
SNR	Signal-to-Noise-Ratio
SVM–RBF	Support Vector Machine with Radial Basis Function kernel
